# Discovery of inhibitors of the cancer-promoting phosphatase PRL-3 and their evaluation in intestinal organoids^☆^

**DOI:** 10.1016/j.bmc.2025.118412

**Published:** 2025-09-23

**Authors:** Andreas Hoffmann, Judith Weyershaeuser, Yamini Chand, Raphael Geißen, Nico Höfflin, Birgit Hoeger, Maja Köhn

**Affiliations:** a Faculty of Biology, University of Freiburg, 79104 Freiburg, Germany; b Signalling Research Centres BIOSS and CIBSS, University of Freiburg, 79104 Freiburg, Germany; c Institute for Cell Biology, University of Bonn, 53115 Bonn, Germany; d Chemical Biology Program, Sloan Kettering Institute, Memorial Sloan Kettering Cancer Center, New York, NY 10065, United States; e Genome Biology Unit, EMBL Heidelberg, 69117 Heidelberg, Germany

**Keywords:** Phosphatase, Cancer, Small molecule inhibitors, Organoids, PRL-3, Compound stability, Molecular docking

## Abstract

Upregulation of the phosphatase of regenerating liver (PRL)-3 is associated with colorectal cancer as well as metastasis development and progression. PRL-3’s overexpression impairs intestinal self-renewal capacity through causing intestinal stem cell death, correlating this cellular mechanism of tumor onset with PRL-3-mediated higher susceptibility to tumor formation upon inflammatory or mutational events. Therefore, PRL-3 inhibitors hold promise as potential therapeutic agents for cancer treatment. Based on the structure of the PRL inhibitor Analog 3, we evaluated here two sets of focused small molecule libraries and screened them in order to identify more potent and selective PRL-3 inhibitors. The best hit, named PRLthiophenib, showed higher inhibition potency *in vitro* than Analog 3 with improved selectivity for PRL-3 over other protein tyrosine phosphatases (PTPs), but not within the PRL family, which continues to be a challenge. PRLthiophenib presented high cellular stability and no long-term cytotoxicity. Furthermore, it rescued the growth capacity of an inducible PRL-3-expressing three-dimensional intestinal cell culture organoid system derived from a PRL-3 overexpressing mouse line, mimicking the rescue of intestinal self-renewal capacity. The results introduce PRLthiophenib as a complementary inhibitor to published ones and support drug discovery efforts toward therapeutic targeting of this challenging cancer-promoting phosphatase.

## 1. Introduction

Phosphatase of regenerating liver-3 (PRL-3) belongs to the family of protein tyrosine phosphatases (PTPs) and is of therapeutic interest due to its implication in tumorigenesis and metastasis formation. High levels of PRL-3 expression lead to enhanced proliferation, migration, and tumor growth.^1–4^ In addition, PRL-3 expression imparts a higher susceptibility to tumor formation upon inflammatory or mutational events in the colon.^5^ This correlates with the reduction of intestinal self-renewal capacity mediated by PRL-3 activity, which is a mechanism for tumor onset.^6,7^ Its full cellular functions and physiological protein substrates are still under investigation. To this end, being able to selectively modulate the catalytic activity of PRL-3 is essential to study its molecular mechanisms and natural substrates. At the same time, PRL-3 represents an attractive drug target for cancer therapy and drug development.^1–4^ In this regard, therapeutic antibodies have shown a remarkable effect on PRL-3-mediated cancer, despite its intracellular localization.^8,9^ Mechanistically, it was suggested that PRL-3 can be externalized from cells and recognized by antibodies, and that the antibodies potentially recruit immunocytes to tumor microenvironments that then kill cancer cells.^8^ However, in general their application is rather inconvenient and expensive compared to an orally available small molecule-based therapy. The same applies for the use of PRL-3 specific nanobodies.^10^

**Fig. 1.**
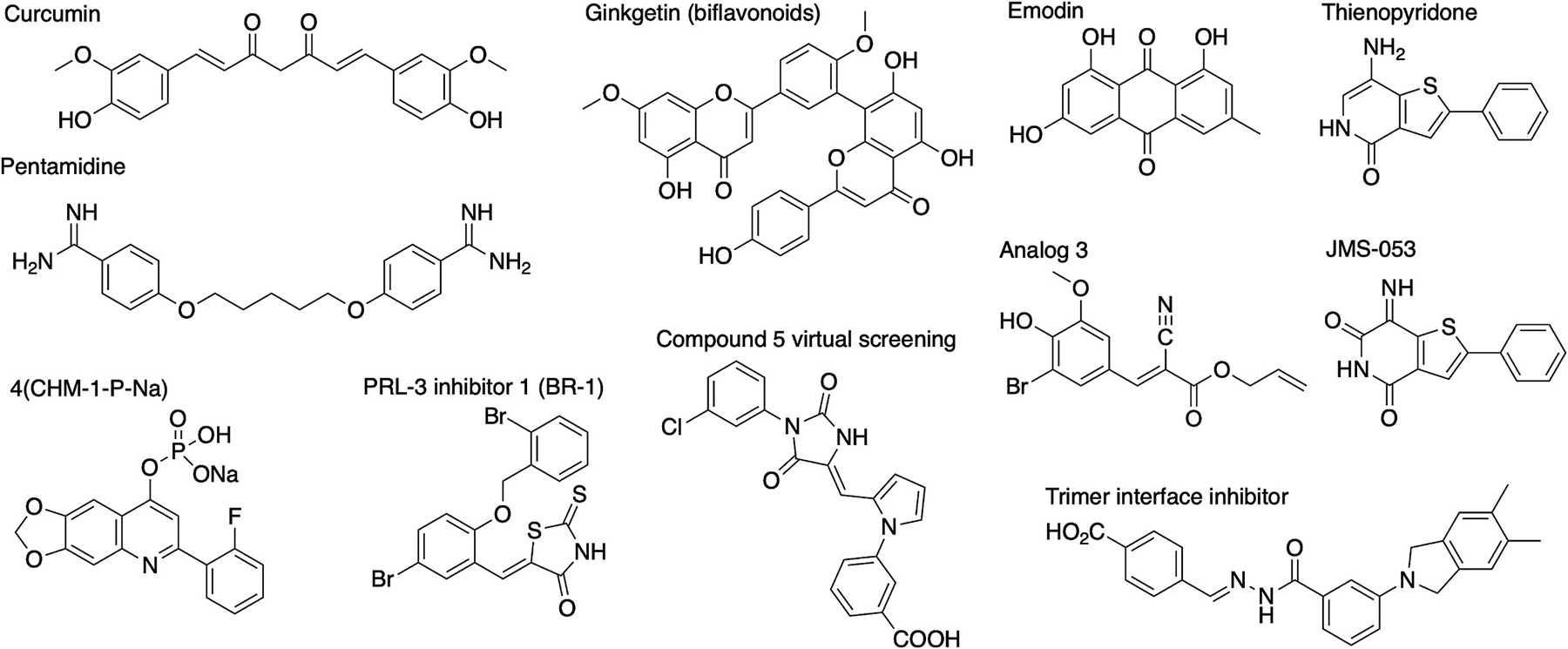
Examples of reported inhibitors of the PRLs.^15–25^

PTP drug development in general is difficult due to limited selectivity and poor pharmacokinetics displayed by traditional active site inhibitors. One of the main limitations is that active site inhibitors have to be negatively charged (mimicking the phosphate group) and therefore show poor bio-availability.^11–14^ Moreover, the development of specific PRL-3 inhibitors is challenging due to the high sequence identity of PRL-3 with its other family members PRL-1 and PRL-2 (79 % with PRL-1, 76 % with PRL-2).^1,11^ In addition, PRLs are small single domain proteins with a size around 20 kDa and exhibit no regulatory domains, leaving little room for mechanism-based allosteric inhibitors.^1,11^ In this regard, Zhang and colleagues developed an inhibitor that blocks the trimerization of all three PRL phosphatases (Fig. 1).^15^ Also the shallow and hydrophobic active site makes it difficult to identify inhibitors selectively targeting PRL-3.^1,11^

There are several small molecule-based PRL inhibitors already published (Fig. 1), but they are not yet selective for PRL-3 – particularly within the PRL family.^15–25^ For example, thienopyridone was one of the first potent PRL inhibitors, found through high-throughput screening. It showed a half maximal inhibitory concentration (IC_50_) of 128 nM for PRL-3, 173 nM for PRL-1 and 277 nM for PRL-2.^17^ Its improved analog, JMS-053, exhibited IC_50_s of 35 nM for PRL-3, 29 nM for PRL-1 and 48 nM for PRL-2.^18^ Nevertheless, it was suggested that both, thienopyridone and JMS-053, oxidize the catalytic cysteine of PTPs and thereby display unspecific inhibition,^21,24^ although this is disputed for JMS-053.^18^ Accordingly, thienopyridone showed strong PRL-3-independent effects in causing cellular long-term toxicity.^24^ As such, there is a continued interest to identify selective, stable, and potent PRL-3 inhibitors.

**Fig. 2.**
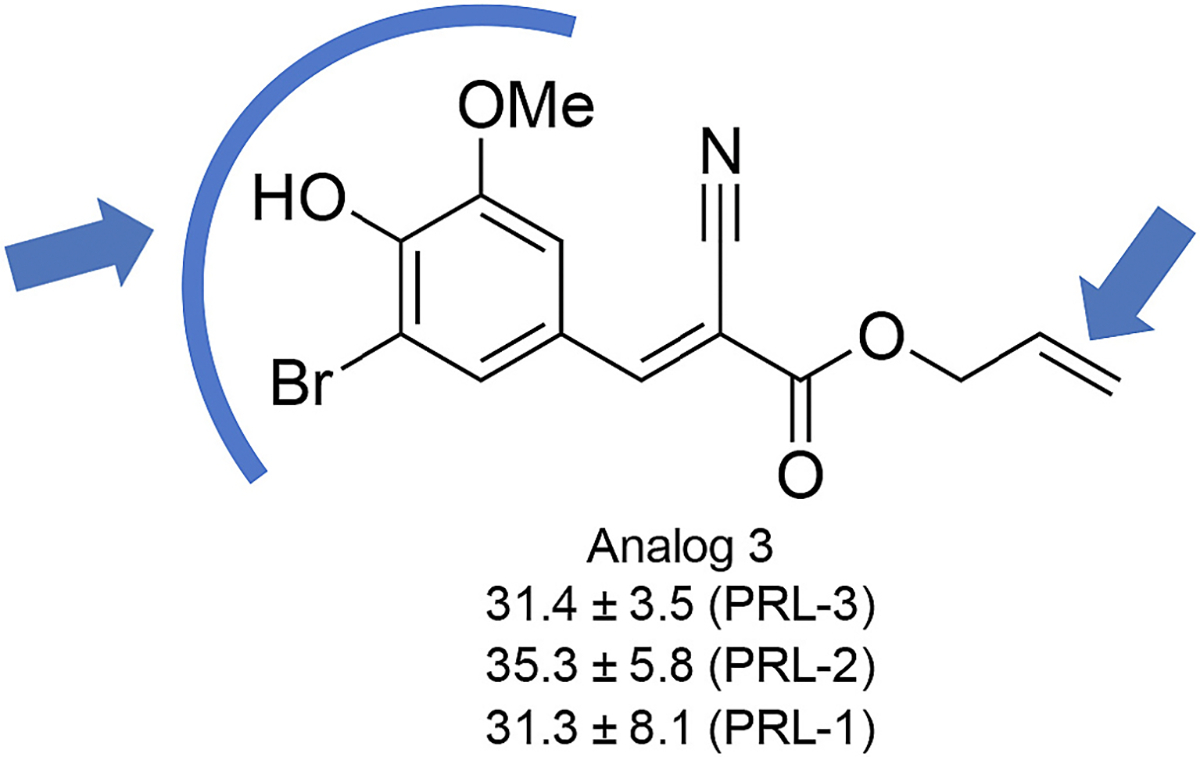
**Structure of Analog 3 with modification points** for structure-activity relationship studies of chemical moieties (indicated with arrows). IC_50_ values (μM) for inhibition of DiFMUP dephosphorylation are shown for PRL-3, PRL-2 and PRL-1.^24^

Based on thienopyridone we previously developed small-molecule PRL inhibitors using an ultrafast shape recognition (USR) method for the detection of molecules with similar pharmacophore features. This computational approach led to the discovery of a 2-cyano-2-ene-ester, a new inhibitor chemotype for PRLs. After a subsequent structure-activity-relationship (SAR) study, Analog 3 was identified (Fig. 1). It is a non-competitive, cell-active PRL inhibitor, demonstrating acceptable selectivity over other PTPs. Analog 3 inhibition toward PRL-3 is not as potent compared to thienopyridone, but in contrast it neither caused cell proliferation inhibition nor underwent oxidation. Additionally, Analog 3 inhibited the catalytic PRL-3 induced phenotype in cellular migration assays as well as in two phenotypically different 3D cell culture models.^6,24,26,27^ However, Analog 3 showed equal inhibition for all PRLs (IC_50_ are 31.4 μM for PRL-3, and 31.3 μM for PRL-1 and 35.3 μM for PRL-2; Fig. 2).^24^

In this study, we aimed to improve Analog 3 in order to identify more potent and selective PRL-3 inhibitors. To this end, we applied traditional SAR studies on the structure of Analog 3 and screened two generations of analogs for PRL-3 inhibition *in vitro.* The most promising candidates were then tested *in vitro* for potency and selectivity over other PTPs. One compound, which was named PRLthiophenib, was selected and assessed for its cellular stability in comparison to the most commonly used inhibitor JMS-053. We found JMS-053 to be degraded very quickly, while PRLthiophenib was very stable. PRLthiophenib was then studied in a PRL-3-dependent three-dimensional (3D) cellular *ex vivo* context and compared to Analog 3 as well as JMS-053. This 3D-cellular primary intestinal organoid-based method mimics the rescue of intestinal self-renewal capacity based on intestinal stem cell (ISC) survival as a specific response to PRL-3 inhibition.^6^ The small intestinal (SI) organoids were derived from a transgenic inducible PRL-3 mouse line with healthy, non-oncogenic background.^6^ Other published 3D-cellular methods to test PRL inhibitors are based on preventing the formation and proliferation of oncogenic spheroid growth. However, it is unclear if this inhibition is a direct effect on PRL-3 due to the general cell growth inhibition phenotype displayed upon inhibitor treatment in cancer cells with complex genetic background.^18^ On the contrary, the death of SI organoids is a direct consequence of PRL-3 expression, without the influence of other complex oncogenes as the genetic background is from a healthy mouse.^6^ As such, the rescue of cell death by an inhibitor is a direct action on PRL-3 and PRL-3-driven mechanisms. Furthermore, organoids are more sensitive and *in vivo*-like compared to spheroids.^28–30^ Therefore, the rescue of PRL-3-induced SI organoid death is a highly PRL-3-specific and biologically relevant read-out, in particular when compared to the artificial PRL-3 overexpressing HEK293-cell migration assays.^24^ We exploited this unique cellular PRL-3-mediated phenotype to assess our compounds for cellular PRL-3 inhibition ability as well as general cytotoxic effects of the compounds. Analog 3 and its derivative PRLthiophenib, but not JMS-053, rescued this PRL-3-induced phenotype with no cytotoxic side effects.

## 2. Results

### 2.1. Structure-activity relationship study based on analog 3

In order to find more potent and specific PRL-3 inhibitors, we performed structure-activity relationship (SAR) analysis on the structure of Analog 3, keeping the inhibitory beneficial structural elements. We previously found that the 2-cyano-2-ene structure was essential for the activity of Analog 3. A hydroxyl group in position 2 of the aromatic ring of Analog 3 and a methoxy group in position 3 was beneficial but not essential for inhibition.^24^ Exchanging the small residues on positions 2 and 3 on the aromatic ring of Analog 3 to bulkier ones, including bulkier ethers, or omitting them led to a reduced activity.^24^ Based on this prior knowledge we aimed here at investigating the effects of the nature of the ester and the substitution pattern as well as the halogen substitution on the aromatic ring (Fig. 2). We also included different bulky residues at position 1 (Br in Analog 3).

**Scheme 1.**
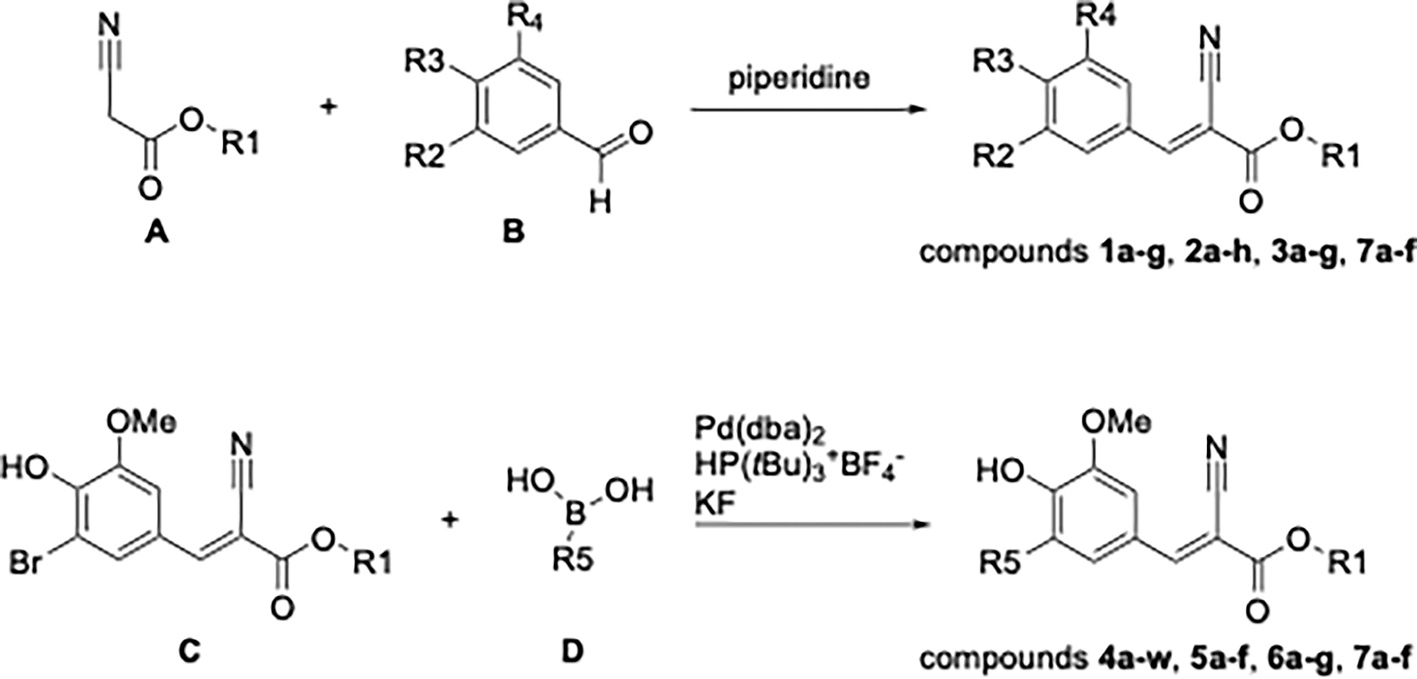
General synthesis scheme for the SAR study. Compounds were synthesized and provided by the Chemical Biology Core Facility at EMBL.

The synthesis of the 1st generation library involved 2 steps (Scheme 1): 1) the condensation of cyano acetates **A** with different aldehyde building blocks **B** (compounds **1a-g; 2a-h; 3a-g**; Table 1 entries 1–22) Suzuki coupling of compound **C** with different boronic acids **D** (compounds **5a-f**; **6a-h**; Table 1 entries 23–59) (Scheme 1). The 2nd generation of compounds, which was designed after retrieving the screening results of the 1st generation, were synthesized using a combination of the condensation to introduce different esters, and then of the Suzuki coupling to introduce the acetyl thiophene residue (compounds **7a-f**; Table 1 entries 60–70).

The compounds were screened *in vitro* for inhibition against human recombinant PRL-3 using the artificial phosphorylated substrate 6,8-difluoro-4-methylumbelliferyl phosphate (DiFMUP) (Table 1). Initially, the substitution pattern on the phenyl ring was altered and Br was replaced by Cl, H, methyl or F. Three different esters were tested in this round: the allyl ester as present in Analog 3 (Table 1, entries 1–7), the benzyl ester (Table 1, entries 8–15), and the 2-methoxy-ethylester (Table 1, entries 16–22). In the first set, only the compound (**1c**) where Br was replaced with F remained weakly active (Table 1, entry 3). When the allyl ester was replaced with the benzyl ester, the same substitution pattern in the benzyl ring as in Analog 3 showed no difference in IC_50_ compared to Analog 3 (**2a**, entry 8), and the Br replacement with Cl (**2c**) and F (**2d**) also led to a retained potency, albeit lower than Analog 3 (entries 10 and 11, respectively). In the set of compounds carrying the 2-methoxy-ethylester only the one with the same substitution pattern at the benzyl ring as Analog 3 remained active, but also to a lesser extent (**3a**, entry 16). This narrow SAR is in agreement with the results from the first optimization of Analog 3, where also very few analogs were found to be active.^24^

Since the benzyl ester-substituted compounds showed the most flexibility with regards to different substitutions at the bromo position, we kept the benzyl ester constant for the next set of compounds and tested different replacements through Suzuki-coupling at the bromo position at the benzyl ring (Table 1, entries 23–59). Again, in general the SAR was very narrow, and most compounds were inactive. Remarkably, two compounds (entries 49 and 50; **5d** and **5e**) showed a higher inhibitory effect than Analog 3. In compound **5d** (entry 49), the bromo group was substituted by a 2-acetylthiophene group and in compound **5e** by a 2-cyanothiophene group (entry 50). As a first follow-up, we tested the effect of the compounds on cytotoxicity. The cyanothiophene-containing compound (**5e**) showed cytotoxicity (Supporting Fig. S1), and therefore second-generation SAR studies were based on compound **5d**.

For the second SAR generation, the benzyl ester of the thiophene-containing compound (**5d**) was modified (Table 1, entries 60–70). In contrast to the first generation, all compounds of the second generation showed similar or better IC_50_ values than Analog 3 toward PRL-3. However, just a few showed lower IC_50_ values than the precursor (**5d**). Compound **7b** (entry 61) with an IC_50_ of 6 μM contained a chloro substitution in para-position of the benzyl ester, compound **7f** (entry 65) with a similar IC_50_ a methoxy substitution in ortho-position, compound **7h** (entry 67) with an IC_50_ of 7.8 μM had a methyl group in ortho-position, and compound **7k** (entry 70) with an IC_50_ of 9.2 μM carried a thiophene instead of the phenyl ester. Due to their low IC_50_ values, compounds **7b**, **7f**, **7h** and **7k** (in the following referred to as *hit compounds*) were interesting candidates and were therefore further characterized.

### 2.2. Selectivity of hit compounds toward different phosphatases

In order to assess potential side effects, the off-target selectivity of the compounds toward other recombinant phosphatases was examined. The residual phosphatase activity was measured after treatment with Analog 3 or the hit compounds (Table 2). PRL-3ś residual phosphatase activity was compared to the close homologs PRL-1 and PRL-2. Additionally, *Vaccinia* H1-related (VHR) phosphatase, selected due to its close structural similarities with the PRL family,^31^ and protein tyrosine phosphatase 1B (PTP1B) and T cell protein tyrosine phosphatase (TCPTP) as representative members of the classical PTPs^24^ were compared (Table 2).

Again, the *in vitro* DiFMUP assay was used. To achieve nearly full inhibition of PRL-3, all compounds were used at the concentration of 30 μM. Enzyme activity was normalized by adjusting the DiFMUP substrate concentration to the enzymeś*K*_*m*_ values. Due to higher activity of the other PTPs compared to the much less active PRLs, the enzyme concentration of the PTPs was set to a comparable activity (PTPs: 0.5 nM; PRL-3: 50 nM), as done previously.^24^ The lower enzyme activity results from variations of amino acids in the active site in the PRLs compared to the canonical residues in other PTPs.^31^

The different PRL enzymes did not show the same activity, even though their different *K*_*m*_ values were taken into account. After an enzyme titration was performed (Supporting Fig. S2), the concentration of PRL-1 and PRL-2 proteins was increased to obtain an equal activity compared to PRL-3 (PRL-1: 150 nM; PRL-2: 100 nM; PRL-3: 50 nM).

The hit compounds **7b** and **7h** fully inhibited PRL-3. PRL-3 activity was decreased by hit compounds **7f** and **7k** down to 10 %, whereas Analog 3-treated PRL-3 still showed around 45 % enzyme activity (Table 2). This reflects the lower IC_50_ of the hit compounds compared to Analog 3 (Table 1). All inhibitors showed specificity for PRL-3 over the other PTPs. VHR, PTP1B and TCPTP still showed at least 50 % phosphatase activity after compound treatement, except VHR, which exhibited 42 % residual phosphatase activity for compound **7b**.

The hit compounds showed stronger specificity toward PRL-3 than Analog 3, except for compound **7b**, which inhibited also PRL-1 and -2 considerably. With compounds **7f**, **7h** and **7k**, PRL-1 and PRL-2 showed at least 38 % phosphatase activity. Overall, compound **7h** performed best regarding selectivity for PRL-3 in this assay. To corroborate this data, we measured the IC_50_ values of compound **7 h** for PRL-1 and PRL-2 (Table 1, entry 69; Supporting Fig. S3). Also here, a trend concerning a stronger potency of compound **7h** against PRL-3 was confirmed.

### 2.3. Short- and long-term cytotoxicity of hit compounds

**Fig. 3.**
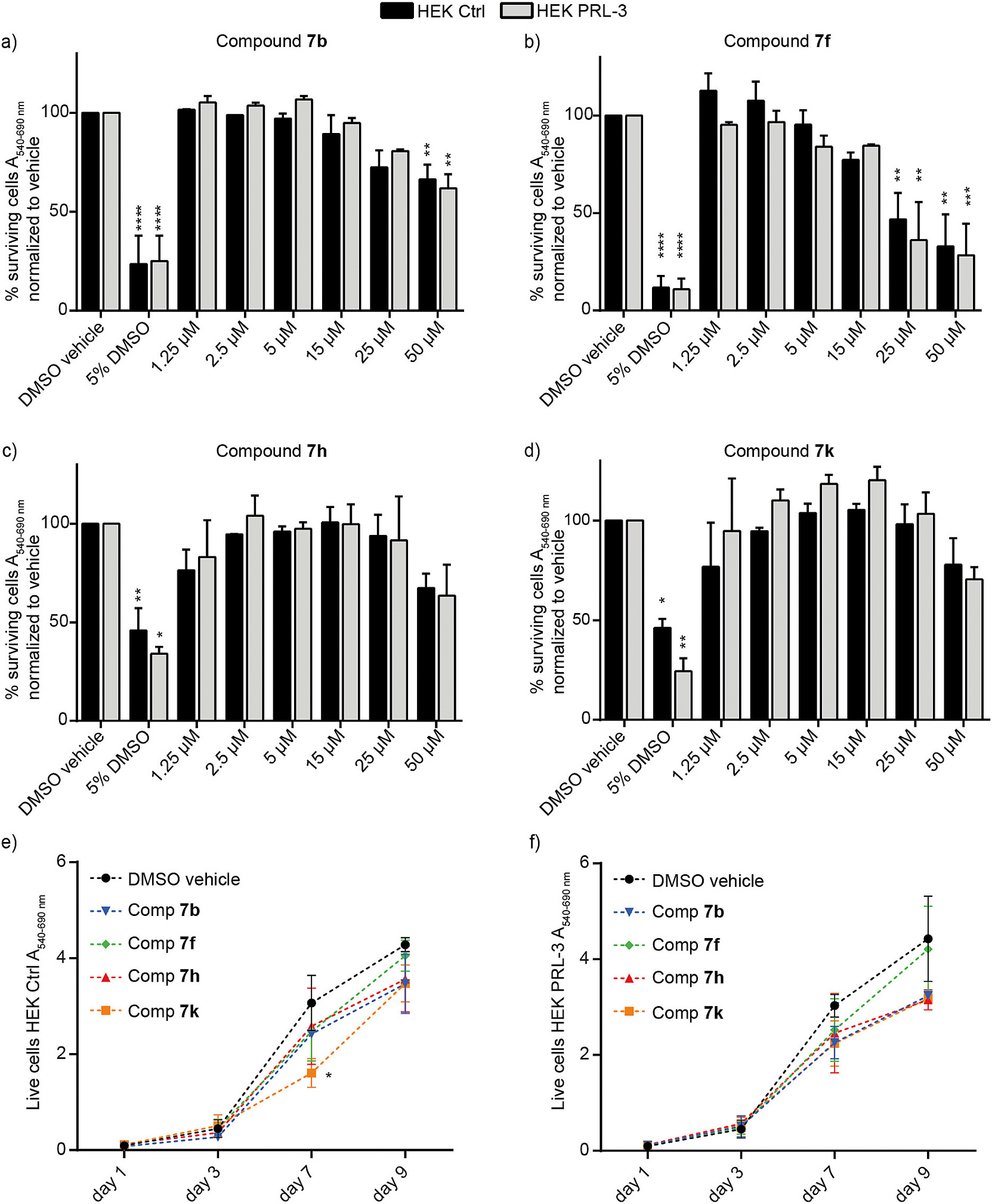
Cytotoxicity of hit compounds. Short-term cytotoxicity of respective hit compounds (a-d) with doxycycline-induced (1 μg/mL) PRL-3 expressing (grey) or empty vector (black) HEK293 cell lines. After 16 h incubation, surviving cells were stained with MTT and absorbance was normalized to DMSO vehicle. Results are depicted as mean + SEM, with compounds concentrations as indicated. One-way ANOVA tests were performed compared to DMSO vehicle. Experiments were performed at least two times in triplicates. Long-term cytotoxicity of respective hit compounds at 10 μM with dox-induced (1 μg/mL) empty vector (e) or PRL-3 expressing (f) HEK293 cell lines. MTT staining and viability was determined at each indicated day. Results are depicted as mean ± SEM. Two-way ANOVA test was performed for *p* < 0.05 compared to DMSO vehicle. Both experiments were performed at least two times in triplicates.

As the discovered compounds had not yet been tested in cellular approaches, it was important to assess any cytotoxic effects. To evaluate suitable compound concentrations for further cellular experiments, short- and long-term cytotoxicity of the compounds was determined using a 3-(4,5-dimethylthiazol-2-yl)-2,5-diphenyltetrazolium bromide (MTT) assay to measure cell viability (Fig. 3). To determine short-term cytotoxicity, inducible PRL-3-expressing or empty vector control HEK293 cells were treated with MTT after 16 h incubation with the respective compound at different concentrations (Fig. 3a–d).

Significant short-term cytotoxicity could not be observed for compound **7h** and **7k** (Fig. 3c, d). Compound **7f** was cytotoxic at 25 μM and higher concentrations (Fig. 3b). Compound **7b** showed short-term cytotoxicity at 50 μM. For both compounds the cytotoxicity was independent of PRL-3 overexpression.

Because thienopyridone had shown proliferative side effects in earlier studies,^24^ a long-term cytotoxicity analysis of the hit compounds was also done to exclude additional effects not detected by the short-term studies. For this, inducible PRL-3-expressing or control (empty vector) HEK293 cells were treated with 10 μM of hit compounds for up to nine days (Fig. 3e, f). No significant long-term cytotoxicity could be observed for the hit compounds at this concentration after nine days. Compound **7k** displayed slight cytotoxicity in the control HEK293 cells after 7 days, but not after 9 days (Fig. 3e), thus the cells seem to be able to cope with any cytotoxic effects over time at this concentration.

Since compound **7h** displayed the most inhibitory specificity for PRL-3 showing no cytotoxicity at the same time (Tables 1 and 2, Fig. 3), in the following we focused on this compound. Furthermore, we renamed it as PRLthiophenib, based on its inhibitory activity against the PRLs and its important thiophene residue.

### 2.4. PRLthiophenib (7h) binds directly to PRL-3

**Fig. 4.**
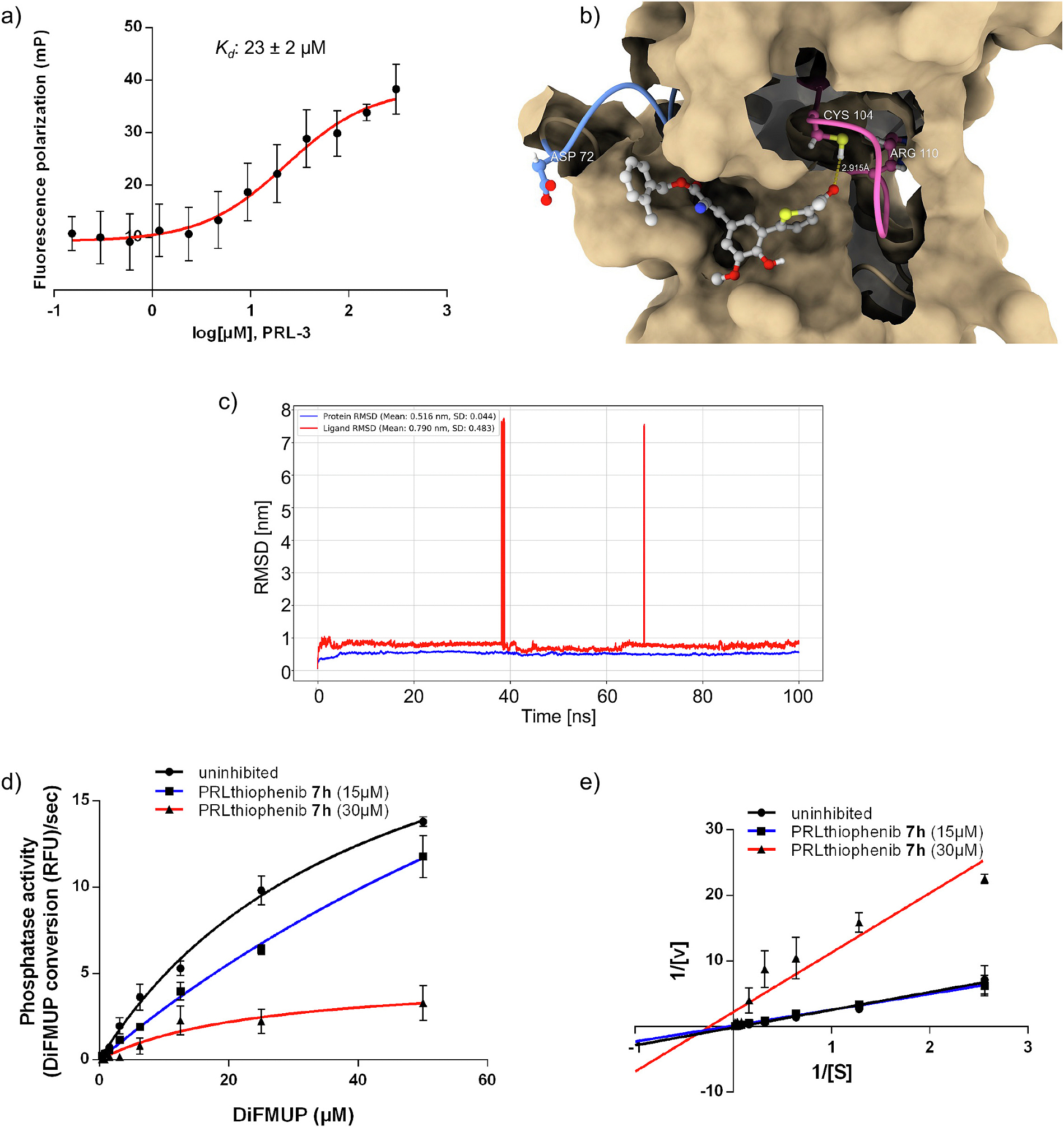
Binding of PRLthiophenib (7h) to PRL-3. (a) Fluorescence polarization saturation binding curve of PRLthiophenib at 10 μM incubated with serial dilutions of recombinant PRL-3 protein (0.15 to 300 μM). Experiment was carried out in three independent experiments in duplicates. Results are depicted as mean ± SEM. The dissociation constant (Kd) was determined by fitting the data to a one-site binding model using GraphPad Prism software (version 6.0). (b) Most stable binding pose of PRLthiophenib in PRL-3 determined by molecular docking.^34^ PRL-3 in surface representation (beige); P-loop (pink; residues 103–110), which includes the nucleophilic Cys104 and Arg110; WPD-loop (blue; residues 68–72), containing the catalytic Asp72. PRLthiophenib is rendered in ball-and-stick format. Visualization was performed using ChimeraX.^35^ (c) RMSD evolution of PRL-3 (blue line) and PRLthiophenib (red line) during a 100 ns molecular dynamics simulation. (d) Michaelis-Menten and (e) Lineweaver-Burk plots of PRLthiophenib (7h) inhibiting DiFMUP dephosphorylation by PRL-3 (50 nM). Both plots show mixed inhibition behavior. Experiment was carried out in at least three independent experiments in technical duplicates. Results are depicted as mean ± SEM.

To investigate whether PRLthiophenib (**7h**) directly binds to PRL-3, we carried out a binding experiment using fluorescence anisotropy, enabled by the autofluorescence of the compound. This experiment revealed a sigmoidal binding curve, establishing a *K*_d_ of 23 ± 2 μM (Fig. 4a), suggesting direct binding and correlating well with the IC_50_.

Next, molecular modeling was applied to identify a potential binding mode for PRLthiophenib. To this end, the NMR-derived structure of PRL-3 (PDB accession code 1V3A, RCSB.org^32^) was used because it (i) lacks any bound ligand, thus avoiding potential docking bias, (ii) adopts an open conformation suitable for ligand binding, and (iii) was previously employed in our study where we identified Analog 3,^24^ thereby enabling direct comparison with earlier findings. Blind docking^33,34^ identified five potential binding pockets on PRL-3 (C1–C5). Among these, C1 exhibited the most favorable Vina score^34^ and the largest cavity volume, suggesting a strong likelihood of biologically relevant binding (Supporting Table 1). Pockets C2 through C5 displayed less negative Vina scores and notably smaller cavity volumes (Supporting Table 1 and Supporting Fig. 4a–d). Although these pockets may still accommodate PRLthiophenib, their weaker binding scores suggest they function as secondary or peripheral sites. As a result, subsequent analyses focused primarily on the top-scoring site, C1 (Fig. 4b, Supporting Fig. 4e). In this pose, PRLthiophenib occupies a cavity that stretches to the WPD loop that carries Asp72, which is important for catalysis, to the catalytic cysteine. Analog 3 was also found to preferably bind to this pocket in docking studies, with the ability to block Asp72, but did not reach the catalytic Cys104.^24^ In contrast, the 2-acetylthiophene group of PRLthiophenib extends toward Cys104, with its carboxyl group positioned approximately 2.915 Å from the thiol of this catalytic residue (Fig. 4b), consistent with the potential for covalent interaction. While this modeled geometry strongly suggests that Cys104 could be directly engaged, experimental confirmation needs to be performed to verify covalent adduct formation.

To further explore the dynamic behavior and stability of the protein–ligand complex, a 100 ns molecular dynamics simulation was performed. In Fig. 4c, the Root Mean Square Deviation (RMSD) profiles of PRL-3 (blue) and PRLthiophenib (red) are shown. After the initial equilibration phase, the protein reached a stable conformation, with an average RMSD of 0.516 nm (SD = 0.044 nm), indicating that its global fold remains consistent throughout the simulation. The ligand, on the other hand, had a slightly higher average RMSD of 0.790 nm (SD = 0.483 nm), reflecting moderate flexibility within the binding. Two sharp spikes in the ligand RMSD (~7 nm at ~40 ns and ~ 60 ns) indicate transient unbinding events, where the ligand briefly dissociated before returning. Such episodes suggest that the current ligand–pocket interactions are not consistently maintained, which may limit sustained target engagement and, consequently, biological efficacy. Overall, the results highlight the stable nature of the protein fold when bound to the ligand, and the ligand’s general retention within the binding site, despite brief episodes of higher movement.

To biochemically determine the mode of inhibition of PRLthiophenib (**7h**) against PRL-3, a DiFMUP dephosphorylation assay was carried out (Fig. 4d, e) as done previously for Analog 3.^24^ Increasing concentrations of PRLthiophenib lowered the apparent *V*_*max*_ with only a modest increase in *K*_*m*_, as indicated by the slight rightward shift of the Michaelis–Menten curves (Fig. 4d). In the Lineweaver–Burk representation (Fig. 4e), the fitted lines intersected to the left of the y-axis, which is inconsistent with a purely competitive or noncompetitive mechanism. The kinetic pattern is most consistent with mixed-type inhibition, predominantly reducing *V*_*max*_ while moderately affecting *K*_*m*_. Together, the data supports the modeling data in suggesting mixed modes of action, which could include transient unbinding, binding rearrangement between noncompetitive and competitive mode, including possible partial covalent interaction. These observations point to the potential benefit of designing future analogs with improved binding persistence to achieve a more stable engagement of the target pocket.

To assess the flexibility of specific protein regions, Root Mean Square Fluctuation (RMSF) values were calculated over the course of the simulation (Supporting Fig. S5a). As expected, the *N*-terminus shows the highest mobility. Most internal residues, however, exhibit fluctuations below 0.4 nm, consistent with well-defined secondary structures. A moderate peak near residue 60 suggests a more dynamic loop or turn in that region. The *C*-terminus also displays slightly increased flexibility compared to the protein’s core regions. These RMSF findings align with the RMSD data, confirming that the protein fold remains stable over the course of the simulation when bound to the ligand, aside from terminal regions and a small flexible segment in the middle of the sequence. Finally, the Radius of Gyration (Rg) was calculated to examine changes in the overall compactness of the protein (Supporting Fig. S5b). The overall observations suggest that the protein undergoes minor structural adjustments early in the simulation before reaching a more tightly packed state.

### 2.5. Rescue of PRL-3-induced cell death in a three-dimensional PRL-3 expressing organoid system by PRLthiophenib

Next, we tested PRLthiophenib (**7h**) in a three-dimensional PRL-3 organoid system in comparison to the most applied inhibitor JMS-053 as well as Analog 3. For this comparison, we first ensured that JMS-053 is active by confirming its IC_50_ in our *in vitro* dephosphorylation assay as 76 ± 1.2 nM (Supporting Fig. S6).

**Fig. 5.**
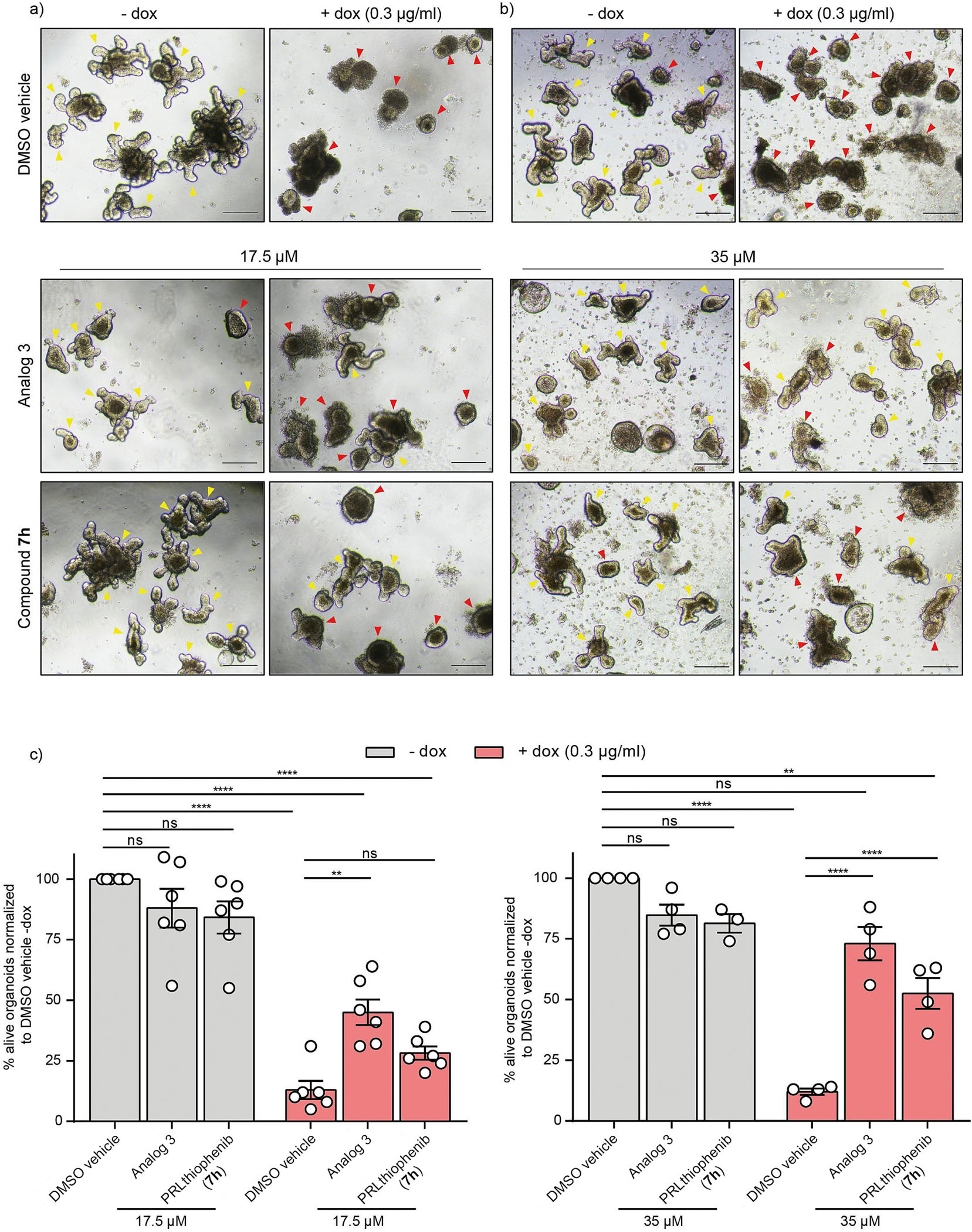
Compounds rescue the apoptotic effect induced by PRL-3 expression in SI organoids. Mouse derived small intestine (SI) organoids containing heterozygous HA-PRL-3 were grown for three days. PRL-3 expression was then induced by doxycycline (dox, 0.3 μg/mL). At the same time SI organoids were treated with 17.5 μM (a) or 35 μM (b) Analog 3 or PRLthiophenib (**7h**) and imaged after 24 h (h). (a, b) Representative bright field images after 24 h of dox and compound- or DMSO vehicle-treated SI organoids indicating dying (red arrows) and alive SI organoids (yellow arrows). Scale bar: 200 μm. (c) Quantification of counted alive SI organoids for both compound concentrations. Results are depicted as mean ± SEM. Two-way ANOVA test was performed for *p* < 0.05. ns: not significant. Experiments were carried out in at least four independent experiments (round data points) with SI organoids derived from at least two mice, all in technical triplicates.

For the cellular assay we exploited a previously described PRL-3-dependent phenotype in which PRL-3 expression kills specifically the stem cells of murine intestinal epithelial organoids.^6^ Primary small intestinal (SI) organoids obtained from a heterozygous (het) PRL-3 doxycycline-inducible overexpressing mouse (HA-PRL-3 het/R26-rtTA het) were let grow for three days to fully develop the characteristic crypt villi-like morphology (Fig. 5a, b). HA-PRL-3 expression was then induced by doxycycline (dox). Analog 3, PRLthiophenib (**7h**) or JMS-053 were added at the same time to rescue any PRL-3-mediated early cell death. The organoids were then imaged after 24 h. Intestinal stem cells (ISCs) are located in the crypts, which represent the tip of the SI organoids’ branches (yellow arrows in Fig. 5a, b). As expected,^6^ upon PRL-3 expression (+ dox) SI organoids lost their crypt villi-like morphology, displayed by getting rounder and exhibiting darker contrast (see red arrows in Fig. 5a, b). The SI organoid images were then analyzed manually by classifying and quantifying the different phenotypes (Fig. 5c).

**Fig. 6.**
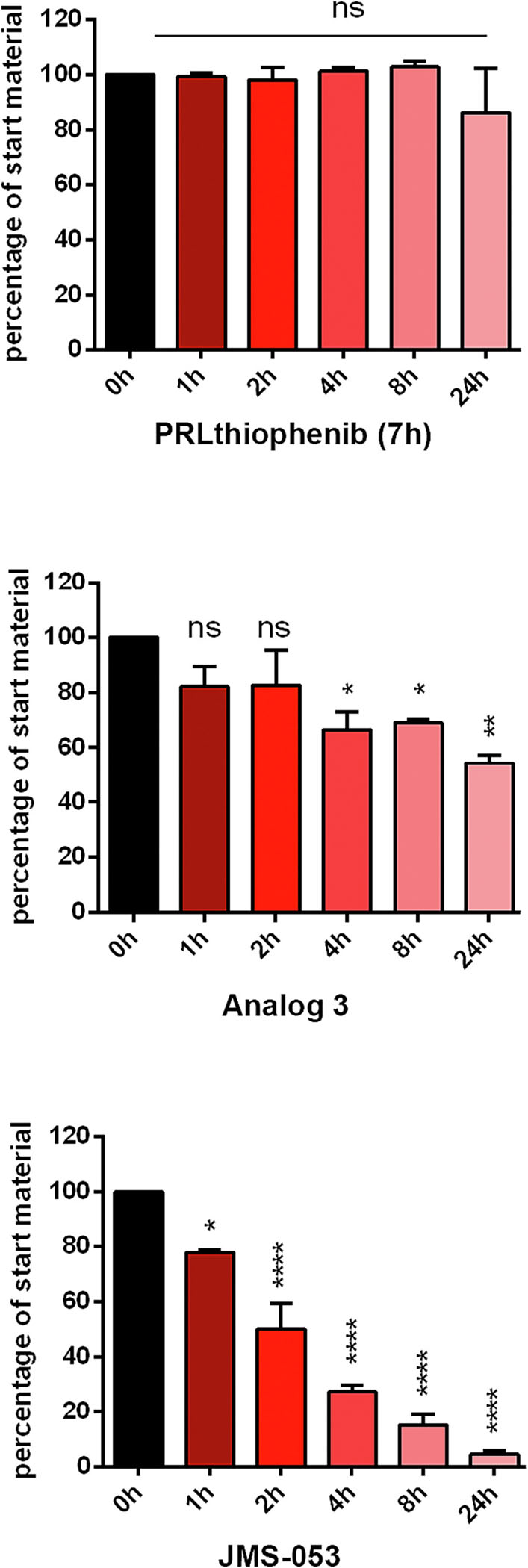
Stability of compounds (100 μM) in HeLa cell lysate. At each time point remaining percentage of start material was measured *via* a solid-phase extraction mass spectrometry (SPE-MS/MS) assay. The experiment was carried out in three independent experiments in triplicates. The results are depicted as mean ± SEM, one-way ANOVA with post-hoc Tukey test was applied compared to 0 h time point.

Both, Analog 3 and PRLthiophenib, prevented the PRL-3-induced ISC death significantly at 35 μM concentration compared to the DMSO vehicle control (Fig. 5b, c). Also, at half the concentration (17.5 μM) Analog 3 rescued the PRL-3-induced phenotype significantly. While not statistically significant, the viability in induced PRLthiophenib-treated SI organoids was increased over 50 % compared to induced vehicle-treated SI organoids (Fig. 5a, c). In general, Analog 3 showed slightly better rescue than PRLthiophenib (Fig. 5c), possibly due to a more efficient uptake given its smaller size. Interestingly, both PRLthiophenib and Analog 3 showed no significant cytotoxicity toward the primary SI organoids at both concentrations (17.5 and 35 μM) in the uninduced (–dox) control cells, corroborating the results from the cytotoxicity assays (Fig. 3) and highlighting the value of this assay rescuing cell death in sensitive primary organoids from a non-cancerous background, enabling to assess cytotoxicity at the same time as efficacy of the compounds. While JMS-053 did not show any cytotoxicity either, we neither observed any rescue of ISC death when tested at the previously described efficacious concentration of 5 μM^36^ (Supporting Fig. S7). At higher concentration (17.5 μM), JMS-053 treatment showed cytotoxicity in organoids, and no rescue of cell death (Supporting Fig. S7). To test whether the inactivity at the non-cytotoxic, previously described as cellular active concentration could be due to a lesser compound stability, we incubated all three compounds in HeLa cell lysates (due to technical feasibility) and measured their presence over time by mass spectrometry. Indeed, JMS-053 was degraded in a matter of a few hours, with almost no detectable compound left after 24 h (Fig. 6). At that time point, which correlates with the organoid incubation time, Analog 3 was still present to about 50 %, whereas PRLthiophenib was not degraded to a statistically significant amount. Nonetheless, JMS-053 was shown to be potent in reducing ovarian cancer cell growth in mice upon *in vitro* pretreatment of the cells with JMS-053 prior to their injection into the mice, followed by a daily regimen administered to the mice.^36^ This shows that while the compound stability might be less optimal and requires repeated dosing, this compound is active in a different context than tested here. Together, these results support that while having a higher IC_50_ *in vitro*, the higher stability of Analog 3 and PRLthiophenib is beneficial in this *ex vivo* experimental setting.

## 3. Conclusion

In summary, we discovered small-molecule compounds with IC_50_s in the low micromolar range for PRL-3 inhibition (Table 1), displaying higher *in vitro* potency compared to Analog 3. While these PRL inhibitors are not as potent *in vitro* as thienopyridone or JMS-053, we showed for **7h** that it is more stable in cell lysates and not long-term cytotoxic (Fig. 3e, f) in contrast to thienopyridone.^24^ Even in the sensitive primary organoid system Analog 3 and **7h** did not show short-term cytotoxicity at 10 μM (Fig. 5).

Most of the four selected candidates (**7f**, **7h** and **7k**) showed good *in vitro* selectivity for PRL-3 over other PTPs (Table 2). The most promising candidate (**7h**) was named PRLthiophenib. Limitations of this compound are that the IC_50_ could not be improved to reach the submicromolar IC_50_ range, and that clear selectivity within the PRL family was still not achieved. Nevertheless, it demonstrated a trend toward *in vitro* selectivity for PRL-3 within the PRL family, which could possibly be explored in the future. It directly binds to PRL-3, likely in the active site pocket (Fig. 4). Furthermore, PRLthiophenib rescued the PRL-3-dependent death phenotype in organoids (Fig. 5). While Analog 3 showed a slightly better rescue than PRLthiophenib, the observed trend regarding the selectivity of PRLthiophenib, higher potency *in vitro* and better *in lysate*-stability (Fig. 6) make it a promising candidate for further improvement. Furthermore, we applied here a sensitive *ex vivo* method to study PRL-3 inhibitors. This method, using inducible PRL-3 mouse SI organoids, offers a clean non-oncogenic background and is based on rescuing cell viability rather than inhibiting proliferation or migration of 2D cells or spheroids with an oncogenic background, giving a clear PRL-3-dependent read-out compared to a cytotoxicity- or migration-based assay in cancerous immortal cells. This method allows to assess the direct PRL-3-dependent phenotype and makes it possible to test cytotoxicity at the same time.

## 4. Experimental section

### 4.1. Compound synthesis

The compounds were synthesized and provided by the Medicinal Chemistry Facility of the Chemical Biology Core Facility at EMBL Heidelberg, Germany, using parallel synthesis and preparative HPLC purification. Yields were not determined for parallel synthesis. Analytical data was provided by the Medicinal Chemistry Facility as well, except for HRMS data of compounds **7a**-**7k** and analytical data of Analog 3, which was measured at the University of Freiburg. Analytical measurements were performed on an Agilent Technologies 1200 series HPLC system with MWD SL UV detector and MS 6120 Single Quadrupole with electrospray ionization (ESI) source. The separation was performed on a reversed phase column (Macherey-Nagel EC 250/4 Nucleodur 100–5 C18ec). High resolution mass spectra were recorded using a MaXis II Q-Tof mass spectrometer (Bruker Daltonics) and at the University of Heidelberg with a Bruker ApexQe hybrid 9.4 T FT-ICR and on a CE-ESI-MS-System (MS: 6520 qTOF-MS, Agilent) at the University of Freiburg. Masses are given as *m*/*z* (% intensity). Nuclear magnetic resonance (NMR) spectra were recorded on a 400 MHz Bruker Avance DPX at EMBL and on a 400 MHz Bruker Avance Neo at the University of Freiburg (Analog 3). All chemicals and anhydrous solvents were obtained from commercial sources (Sigma-Aldrich, VWR, ABCR, Acros) and used without further purification. All final compounds were > 95 % pure by HPLC. For the hit compounds **7b**, **7f**, **7h**, **7k**, and Analog 3, the HPLC, MS and NMR data are provided in the Supporting Information.

General procedure for the parallel synthesis of compounds **1a**-**1g**, **2a**-**2h**, **3a**-**3g** (entries 1–22) and Analog 3:

To a solution of the aldehyde (0.8 mmol) and the cyano acetate (0.8 mmol) in CH_2_Cl_2_ (2 mL) was added piperidine (0.2 eq) and the reaction was shaken at room temperature overnight (18 h). The reaction mixtures were partitioned (10 % *w*/w aq. citric acid/ CH_2_Cl_2_ [2:1 mL]) and filtered through a phase separator which was washed with a further 1 mL CH_2_Cl_2_. A 5 μL sample was taken, dried, diluted with 200 μL of CH_3_CN/H_2_O (1:1) and injected on the LCMS. The CH_2_Cl_2_ was allowed to evaporate from the vials overnight affording crude material. The product was purified by preparative HPLC (Agilent, RP C18. 10–100 % CH_3_CN/H_2_O gradient with 0.1 % TFA. 15-min gradient).

#### Analytical data of the active compounds:

Allyl (*E*)-3-(3-bromo-4-hydroxy-5-methoxyphenyl)-2-cyanoacrylate (**Analog 3**).

^1^H NMR (d6-DMSO, 400 MHz): *δ* = 3.89 (s, 3H), 4.79 (dt, *J* = 5.4, 1.5 Hz, 2H), 5.31 (ddd, *J* = 10.5, 1.4, 1.4 Hz, 1H), 5.42 (ddd, *J* = 17.2, 1.7, 1.7 Hz, 1H), 6.01 (ddt, *J* = 17.2, 10.6, 5.4 Hz, 1H), 7.80 (d, *J* = 2.1 Hz, 1H), 7.99 (d, *J* = 2.0 Hz, 1H), 8.29 (s, 1H), 11.02 (s, 1H). ^13^C NMR (d6-DMSO, 101 MHz): *δ* = 56.76, 66.69, 99.14, 110.14, 113.54, 116.62, 118.86, 123.88, 129.64, 132.43, 148.59, 149.68, 154.38, 162.34. HRMS (ESI neg): [M-H]^−^ calcd. For $C_{14}H_{11}{NO}_{4}^{79}Br$: 335.98769. Found: 335.98789. [M-H]^−^ calcd. For $C_{14}H_{11}{NO}_{4}^{81}Br$: 337.98565. Found: 337.98586.

Allyl (*E*)-2-cyano-3-(3-fluoro-4-hydroxy-5-methoxyphenyl)acrylate (**1c**).

^1^H NMR (d6-DMSO, 400 MHz): *δ* = 3.86 (s, 3H), 4.78 (dt, *J* = 5.4, 1.5 Hz, 2H), 5.30 (ddd, *J* = 10.5, 1.4, 1.4 Hz, 1H), 5.41 (ddd, *J* = 17.2, 1.6, 1.6 Hz, 1H), 6.00 (ddt, *J* = 17.2, 10.7, 5.4 Hz, 1H), 7.64–7.69 (m, 2H), 8.28 (s, 1H), 10.81 (s, 1H). ^13^C NMR (d6-DMSO, 101 MHz): *δ* = 56.32, 66.24, 98.77, 111.15, 112.29 (d, *J* = 20.4), 116.16, 118.41, 121.39 (d, *J* = 9.5 Hz), 131.97, 140.43 (d, *J* = 14.0 Hz), 149.41 (d, *J* = 6.9 Hz), 151.96, 154.36 (d, *J* = 3.1 Hz), 161.90. ^19^F NMR (d6-DMSO, 376 MHz): *δ* = −134.40 (d, *J* = 11.6 Hz). HRMS (ESI neg): [M-H]^−^ calcd. For C_14_H_11_FNO_4_: 276.06776. Found: 276.06789.

Benzyl (*E*)-3-(3-bromo-4-hydroxy-5-methoxyphenyl)-2-cyanoacrylate (**2a**)

^1^H NMR (d6-DMSO, 400 MHz): *δ* = 3.88 (s, 3H), 5.34 (s, 2H), 7.32–7.49 (m, 5H), 7.80 (d, *J* = 2.1 Hz, 1H), 7.99 (d, *J* = 2.0 Hz, 1H), 8.31 (s, 1H), 11.01 (s, 1H). ^13^C NMR (d6-DMSO, 101 MHz): *δ* = 56.32, 67.28, 98.82, 109.67, 113.11, 116.16, 123.44, 128.03, 128.31, 128.55, 129.19, 135.44, 148.14, 149.23, 154.02, 162.14. HRMS (ESI neg): [M-H]^−^ calcd. For $C_{18}H_{13}{NO}_{4}^{79}Br$: 386.00334. Found: 386.00344. [M-H]^−^ calcd. For $C_{18}H_{13}{NO}_{4}^{81}Br$: 388.00130. Found: 388.00149.

Benzyl (*E*)-3-(3-chloro-4-hydroxy-5-methoxyphenyl)-2-cyanoacrylate (**2c**)

^1^H NMR (d6-DMSO, 400 MHz): *δ* = 3.86 (s, 3H), 5.33 (s, 2H), 7.33–7.47 (m, 5H), 7.74 (d, *J* = 2.1 Hz, 1H), 7.83 (d, *J* = 2.1 Hz, 1H), 8.27 (s, 1H), 10.56 (s, 1H). ^13^C NMR (d6-DMSO, 101 MHz): *δ* = 56.24, 67.18, 97.76, 112.54, 116.39, 120.54, 121.97, 126.54, 128.00, 128.28, 128.54, 135.51, 148.69, 149.35, 153.94, 162.34. HRMS (ESI neg): [M-H]^−^ calcd. For C_18_H_13_NO_4_^35^Cl: 342.05386. Found: 342.05403 [M-H]^−^ calcd. For C_18_H_13_NO_4_^37^Cl: 344.05091. Found: 344.05111.

Benzyl (*E*)-2-cyano-3-(3-fluoro-4-hydroxy-5-methoxyphenyl)acrylate (**2d**)

^1^H NMR (d6-DMSO, 400 MHz): *δ* = 3.85 (s, 3H), 5.33 (s, 2H), 7.34–7.49 (m, 5H), 7.63–7.69 (m, 2H), 8.29 (s, 1H), 10.81 (s, 1H). ^13^C NMR (d6-DMSO, 101 MHz): *δ* = 56.29, 67.23, 98.23, 111.18, 112.37 (d, *J* = 20.4), 116.31, 120.96 (d, *J* = 9.4 Hz), 128.01, 128.30, 128.55, 135.48, 141.19 (d, *J* = 15.0 Hz), 149.50 (d, *J* = 6.7 Hz), 152.05, 154.34 (d, *J* = 3.2 Hz), 162.26. ^19^F NMR (d6-DMSO, 376 MHz): *δ* = −134.52 (d, *J* = 11.0 Hz). HRMS (ESI neg): [M-H]^−^ calcd. For C_18_H_13_FNO_4_: 326.08341. Found: 326.08354.

2-Methoxyethyl (*E*)-3-(3-bromo-4-hydroxy-5-methoxyphenyl)-2-cyanoacrylate (**3a**)

^1^H NMR (d6-DMSO, 400 MHz): *δ* = 3.30 (s, 3H), 3.60–3.65 (m, 2H), 3.88 (s, 3H), 4.37–4.41 (m, 2H), 7.79 (d, *J* = 2.2 Hz, 1H), 7.98 (d, *J* = 2.1 Hz, 1H), 8.28 (s, 1H), 11.00 (s, 1H). ^13^C NMR (d6-DMSO, 101 MHz): *δ* = 56.32, 58.18, 65.10, 69.54, 98.80, 109.68, 113.14, 116.15, 123.42, 129.11, 148.15, 149.20, 153.95, 162.23. HRMS (ESI neg): [M-H]^−^ calcd. For $C_{14}H_{13}{NO}_{5}^{79}Br$: 353.99826. Found: 353.99843. [M-H]^−^ calcd. For $C_{14}H_{13}{NO}_{5}^{81}Br$: 355.99621. Found: 355.99638.

#### Analytical data of the inactive compounds:

Allyl (*E*)-2-cyano-3-(4-hydroxyphenyl)acrylate (**1a**).

^1^H NMR (CDCl_3_, 400 MHz): *δ* = 4.81 (dt, *J* = 5.7, 1.3 Hz, 2H), 5.33 (dq, *J* = 10.4, 1.2 Hz, 1H), 5.44 (dq, *J* = 17.3, 1.5 Hz, 1H), 6.01 (ddt, *J* = 17.2, 10.4, 5.6 Hz, 1H), 6.51 (s, 1H), 6.99 (d, *J* = 8.9 Hz, 2H), 7.97 (d, *J* = 8.8 Hz, 2H), 8.22 (s, 1H).

Allyl (*E*)-3-(3-chloro-4-hydroxy-5-methoxyphenyl)-2-cyanoacrylate (**1b**).

^1^H NMR (CDCl_3_, 400 MHz): *δ* = 4.01 (s, 3H), 4.82 (dt, *J* = 5.7, 1.3 Hz, 2H), 5.33 (dq, *J* = 10.6, 1.3 Hz, 1H), 5.44 (dq, *J* = 17.2, 1.3 Hz, 1H), 6.00 (ddt, *J* = 17.1, 10.4, 5.7 Hz, 1H), 6.44 (s, 1H), 7.45 (d, *J* = 2.0 Hz, 1H), 7.80 (d, *J* = 1.9 Hz, 1H), 8.10 (s, 1H).

Allyl (*E*)-2-cyano-3-(4-hydroxy-3-methoxy-5-methylphenyl)acrylate (**1d**)

^1^H NMR (CDCl_3_, 400 MHz): *δ* = 2.28 (s, 3H), 3.97 (s, 3H), 4.81 (dt, *J* = 5.7, 1.3 Hz, 2H), 5.32 (dq, *J* = 10.4, 1.2 Hz, 1H), 5.44 (dq, *J* = 17.2, 1.3 Hz, 1H), 6.01 (ddt, *J* = 17.2, 10.4, 5.7 Hz, 1H), 6.33 (s, 1H), 7.27 (d, *J* = 2.0 Hz, 1H), 7.74 (d, *J* = 2.0 Hz, 1H), 8.10 (s, 1H).

Allyl (*E*)-3-(3-bromo-5-hydroxy-4-methoxyphenyl)-2-cyanoacrylate (**1e**)

^1^H NMR (CDCl_3_, 400 MHz): *δ* = 4.00 (s, 3H), 4.81 (dt, *J* = 5.7, 1.3 Hz, 2H), 5.34 (dq, *J* = 10.5, 1.2 Hz, 1H), 5.44 (dq, *J* = 17.2, 1.5 Hz, 1H), 6.00 (ddt, *J* = 17.2, 10.4, 5.7 Hz, 1H), 7.66 (s, 2H), 8.08 (s, 1H).

Allyl (*E*)-3-(3-bromo-4,5-dimethoxyphenyl)-2-cyanoacrylate (**1f**)

^1^H NMR (CDCl_3_, 400 MHz): *δ* = 3.95 (s, 3H), 3.97 (s, 3H), 4.82 (dt, *J* = 5.7, 1.4 Hz, 2H), 5.34 (dq, *J* = 10.4, 1.2 Hz, 1H), 5.44 (dq, *J* = 17.2, 1.4 Hz, 1H), 6.00 (ddt, *J* = 17.2, 10.4, 5.6 Hz, 1H), 7.59 (d, *J* = 2.1 Hz, 1H), 7.81 (d, *J* = 2.1 Hz, 1H), 8.11 (s, 1H).

Allyl (*E*)-3-(3-bromo-5-methoxyphenyl)-2-cyanoacrylate (**1g**)

^1^H NMR (CDCl_3_, 400 MHz): *δ* = 3.86 (s, 3H), 4.82 (dt, *J* = 5.7, 1.3 Hz, 2H), 5.34 (dq, *J* = 10.4, 1.2 Hz, 1H), 5.45 (dq, *J* = 17.2, 1.4 Hz, 1H), 6.00 (ddt, *J* = 17.2, 10.5, 5.6 Hz, 1H), 7.25 (t, *J* = 1.9 Hz, 1H), 7.56–7.59 (m, 2H), 8.14 (s, 1H).

Benzyl (*E*)-2-cyano-3-(4-hydroxyphenyl)acrylate (**2b**)

^1^H NMR (CDCl_3_, 400 MHz): *δ* = 5.35 (s, 2H), 6.95 (d, *J* = 8.8 Hz, 1H), 7.32–7.46 (m, 5H), 7.96 (d, *J* = 8.8 Hz, 1H), 8.19 (s, 1H).

Benzyl (*E*)-2-cyano-3-(4-hydroxy-3-methoxy-5-methylphenyl)acrylate (**2e**)

^1^H NMR (CDCl_3_, 400 MHz): *δ* = 2.28 (s, 3H), 3.96 (s, 3H), 5.34 (s, 2H), 6.27 (s, 1H), 7.24–7.26 (m, 1H), 7.28–7.47 (m, 5H), 7.73 (d, *J* = 2.1 Hz, 1H), 8.12 (s, 1H).

Benzyl (*E*)-3-(3-bromo-5-hydroxy-4-methoxyphenyl)-2-cyanoacrylate (**2f**)

^1^H NMR (CDCl_3_, 400 MHz): *δ* = 3.99 (s, 3H), 5.35 (s, 2H), 6.10 (s, 1H), 7.33–7.47 (m, 5H), 7.62–7.66 (m, 2H), 8.08 (s, 1H).

Benzyl (*E*)-3-(3-bromo-4,5-dimethoxyphenyl)-2-cyanoacrylate (**2g**)

^1^H NMR (CDCl_3_, 400 MHz): *δ* = 3.94 (s, 3H), 3.96 (s, 3H), 5.36 (s, 2H), 7.32–7.46 (m, 5H), 7.57 (d, *J* = 2.2 Hz, 1H), 7.80 (d, *J* = 2.2 Hz, 1H), 8.11 (s, 1H).

Benzyl (*E*)-3-(3-bromo-5-methoxyphenyl)-2-cyanoacrylate (**2h**)

^1^H NMR (CDCl_3_, 400 MHz): *δ* = 3.85 (s, 3H), 5.36 (s, 2H), 7.23 (t, *J* = 1.9 Hz, 1H), 7.32–7.46 (m, 5H), 7.54–7.57 (m, 2H), 8.13 (s, 1H).

2-Methoxyethyl (*E*)-2-cyano-3-(4-hydroxyphenyl)acrylate (**3b**)

^1^H NMR (CDCl_3_, 400 MHz): *δ* = 3.49 (s, 3H), 3.76–3.79 (m, 2H), 4.46–4.49 (m, 2H), 6.93 (d, *J* = 8.8 Hz, 2H), 7.89 (d, *J* = 8.8 Hz, 2H), 8.04 (s, 1H).

2-Methoxyethyl (*E*)-3-(3-chloro-4-hydroxy-5-methoxyphenyl)-2-cyanoacrylate (**3c**)

^1^H NMR (CDCl_3_, 400 MHz): *δ* = 3.44 (s, 3H), 3.70–3.74 (m, 2H), 4.00 (s, 3H), 4.45–4.48 (m, 2H), 6.53 (s, 1H), 7.44 (d, *J* = 2.0 Hz, 1H), 7.79 (d, *J* = 2.0 Hz, 1H), 8.08 (s, 1H).

2-Methoxyethyl (*E*)-2-cyano-3-(3-fluoro-4-hydroxy-5-methoxyphenyl)acrylate (**3d**)

^1^H NMR (CDCl_3_, 400 MHz): *δ* = 3.44 (s, 3H), 3.70–3.73 (m, 2H), 4.00 (s, 3H), 4.46–4.46 (m, 2H), 6.12 (s, 1H), 7.32 (dd, *J* = 10.5, 2.0 Hz, 1H), 7.60 (s, 1H), 8.08 (s, 1H).

2-Methoxyethyl (*E*)-3-(3-bromo-5-hydroxy-4-methoxyphenyl)-2-cyanoacrylate (**3e**)

^1^H NMR (CDCl_3_, 400 MHz): *δ* = 3.44 (s, 3H), 3.70–3.74 (m, 2H), 4.00 (s, 3H), 4.45–4.49 (m, 2H), 6.04 (s, 1H), 7.64 (s, 2H), 8.07 (s, 1H).

2-Methoxyethyl (*E*)-3-(3-bromo-4,5-dimethoxyphenyl)-2-cyanoacrylate (**3f**)

^1^H NMR (CDCl_3_, 400 MHz): *δ* = 3.44 (s, 3H), 3.70–3.74 (m, 2H), 3.95 (s, 3H), 3.97 (s, 3H), 4.45–4.49 (m, 2H), 7.59 (d, *J* = 2.1 Hz, 1H), 7.80 (d, *J* = 2.1 Hz, 1H), 8.11 (s, 1H).

2-Methoxyethyl (*E*)-3-(3-bromo-5-methoxyphenyl)-2-cyanoacrylate (**3g**)

^1^H NMR (CDCl_3_, 400 MHz): *δ* = 3.44 (s, 3H), 3.69–3.73 (m, 2H), 3.86 (s, 3H), 4.46–4.50 (m, 2H), 7.24 (t, *J* = 2.0 Hz, 1H), 7.56–7.58 (m, 2H), 8.14 (s, 1H).

Larger scale synthesis of benzyl (*E*)-3-(3-bromo-4-hydroxy-5-methoxyphenyl)-2-cyanoacrylate (**2a**) as starting material for the synthesis of compounds **4a**-**4w**, **5a**-**5f**, **6a**-**6h** (entries 23–59):

5-bromovanillin (3,0 g; 0,013 mol), benzyl cyanoacetate (1 eq.; 1.988 mL; 0,013 mol) and piperidine (0,2 eq.; 0,257 mL; 0,003 mol) were dissolved in ethyl acetate (30 mL, reagent grade) and stirred at room temperature overnight. Aqueous work up afforded the crude material as an orange solid (5.00 g; 100 %). Analytical data matched that of the parallel synthesis. The compound was used as starting material for parallel synthesis without further purification.

General procedure for the parallel synthesis of compounds **4a**-**4w**, **5a**-**5f**, **6a**-**6h** (entries 23–59):

Arylbromide **2a** (250 mg; 0.644 mmol), the boronic acid (1.5 eq.; 164.2 mg; 0.966 mmol), Pd(dba)_2_ (8 mol%), tri-*tert*-butyl phosphonium tetrafluoroborate (9.6 mol%) and potassium fluoride (3.3 eq; 123.5 mg; 2.125 mmol) were weighed in and the flask was flushed with argon. Degassed THF (10 mL) was added and the yellow to brown suspension was stirred under reflux overnight. After cooling to room temperature, the mixture was filtered over a small silica pad and washed with ethyl acetate and CH_2_Cl_2_. The filtrate was concentrated under reduced pressure and purified by preparative HPLC (Agilent, RP C18. 20–90 % CH_3_CN/H_2_O gradient with 0.05 % TFA. 35-min gradient).

#### Analytical data of the active compounds:

Benzyl (*E*)-3-(3-(5-acetylthiophen-2-yl)-4-hydroxy-5-methoxyphenyl)-2-cyanoacrylate (**5d**).

^1^H NMR (CDCl_3_, 400 MHz): *δ* = 2.58 (s, 3H), 4.02 (s, 3H), 5.36 (s, 2H), 6.97 (s, 1H), 7.34–7.48 (m, 5H), 7.60 (d, *J* = 3.7 Hz, 1H), 7.69 (d, *J* = 3.8 Hz, 1H), 7.74 (s, 1H), 7.82 (s, 1H), 8.18 (s, 1H). ^13^C NMR (CDCl_3_, 101 MHz): *δ* = 26.91, 56.77, 68.13, 100.3, 110.28, 116.19, 120.27, 124.05, 127.29, 127.41, 128.43, 128.74, 128.85, 132.73, 135.18, 143.93, 145.63, 147.62, 147.74, 154.74, 162.82, 190.97. HRMS (ESI neg): [M-H]^−^ calcd. For C_24_H_18_NO_5_S: 432.09112. Found: 432.09127.

Benzyl (*E*)-2-cyano-3-(3-(5-cyanothiophen-2-yl)-4-hydroxy-5-methoxyphenyl)acrylate (**5e**).

^1^H NMR (CDCl_3_, 400 MHz): *δ* = 4.03 (s, 3H), 5.37 (s, 2H), 7.36–7.48 (m, 5H), 7.56 (d, *J* = 4.0 Hz, 1H), 7.62 (d, *J* = 4.0 Hz, 1H), 7.76–7.80 (m, 2H), 8.19 (s, 1H). ^13^C NMR (CDCl_3_, 101 MHz): *δ* = 56.83, 68.22, 100.79, 109.81, 110.86, 114.52, 116.09, 119.29, 124.29, 126.09, 126.45, 128.46, 128.78, 128.86, 135.11, 137.43, 144.46, 147.26, 147.57, 154.47, 162.70. HR MS (ESI neg): [M-H]^−^ calcd. For C_23_H_15_N_2_O_4_S: 415.07580. Found: 415.07596.

#### Analytical data of the inactive compounds:

Benzyl (*E*)-2-cyano-3-(6-hydroxy-5-methoxy-2′-methyl-[1,1′-biphenyl]-3-yl)acrylate (**4a**).

^1^H NMR (CDCl_3_, 400 MHz): *δ* = 2.19 (s, 3H), 4.02 (s, 3H), 5.34 (s, 2H), 6.28 (s, 1H), 7.16–7.46 (m, 10H), 7.95 (s, 1H), 8.17 (s, 1H).

Benzyl (*E*)-2-cyano-3-(6-hydroxy-5-methoxy-3′-methyl-[1,1′-biphenyl]-3-yl)acrylate (**4b**).

^1^H NMR (CDCl_3_, 400 MHz): *δ* = 2.44 (s, 3H), 4.05 (s, 3H), 5.39 (s, 2H), 6.52 (s, 1H), 7.22 (d, *J* = 7.3 Hz, 1H), 7.34–7.50 (m, 9H), 7.94 (s, 1H), 8.23 (s, 1H).

Benzyl (*E*)-2-cyano-3-(6-hydroxy-5-methoxy-4′-methyl-[1,1′-biphenyl]-3-yl)acrylate (**4c**).

^1^H NMR (CDCl_3_, 400 MHz): *δ* = 2.43 (s, 3H), 4.04 (s, 3H), 5.38 (s, 2H), 6.53 (s, 1H), 7.29 (d, *J* = 7.4 Hz, 1H), 7.36–7.56 (m, 8H), 7.91 (s, 1H), 8.22 (s, 1H).

Benzyl (*E*)-2-cyano-3-(2′-fluoro-6-hydroxy-5-methoxy-[1,1′-biphenyl]-3-yl)acrylate (**4d**).

^1^H NMR (CDCl_3_, 400 MHz): *δ* = 4.00 (s, 3H), 5.34 (s, 2H), 6.21 (s, 1H), 7.15 (t, *J* = 8.8 Hz, 1H), 7.21 (t, *J* = 7.3 Hz, 1H), 7.32–7.45 (m, 8H), 7.93 (s, 1H), 8.18 (s, 1H).

Benzyl (*E*)-2-cyano-3-(3′-fluoro-6-hydroxy-5-methoxy-[1,1′-biphenyl]-3-yl)acrylate (**4e**).

^1^H NMR (CDCl_3_, 400 MHz): *δ* = 4.01 (s, 3H), 5.35 (s, 2H), 5.83 (s, 1H), 7.06 (t, *J* = 8.0 Hz, 1H), 7.30–7.46 (m, 9H), 7.88 (s, 1H), 8.18 (s, 1H).

Benzyl (*E*)-2-cyano-3-(4′-fluoro-6-hydroxy-5-methoxy-[1,1′-bipheny l]-3-yl)acrylate (**4f**).

^1^H NMR (CDCl_3_, 400 MHz): *δ* = 4.01 (s, 3H), 5.35 (s, 2H), 5.83 (s, 1H), 7.12 (t, *J* = 8.7 Hz, 2H), 7.34–7.46 (m, 6H), 7.53–7.58 (m, 2H), 7.85 (s, 1H), 8.18 (s, 1H).

Benzyl (*E*)-2-cyano-3-(6-hydroxy-2′,5-dimethoxy-[1,1′-biphenyl]-3-yl)acrylate (**4g**).

^1^H NMR (CDCl_3_, 400 MHz): *δ* = 3.85 (s, 3H), 4.03 (s, 3H), 5.38 (s, 2H), 6.57 (s, 1H), 7.05 (d, *J* = 8.3 Hz, 1H), 7.10 (d, *J* = 7.4 Hz, 1H), 7.33 (d, *J* = 7.4 Hz, 1H), 7.37–7.50 (m, 7H), 7.94 (s, 1H), 8.22 (s, 1H).

Benzyl (*E*)-2-cyano-3-(6-hydroxy-3′,5-dimethoxy-[1,1′-biphenyl]-3-yl)acrylate (**4h**).

^1^H NMR (CDCl_3_, 400 MHz): *δ* = 3.87 (s, 3H), 4.04 (s, 3H), 5.38 (s, 2H), 6.55 (s, 1H), 6.95 (dd, *J* = 8.2, 2.2 Hz, 1H), 7.16–7.22 (m, 2H), 7.36–7.49 (m, 7H), 7.91 (s, 1H), 8.22 (s, 1H).

Benzyl (*E*)-2-cyano-3-(6-hydroxy-4′,5-dimethoxy-[1,1′-biphenyl]-3-yl)acrylate (**4i**).

^1^H NMR (CDCl_3_, 400 MHz): *δ* = 3.87 (s, 3H), 4.03 (s, 3H), 5.38 (s, 2H), 6.54 (s, 1H), 7.01 (d, *J* = 8.5 Hz, 2H), 7.36–7.49 (m, 6H), 7.56 (d, *J* = 8.6 Hz, 2H), 7.87 (s, 1H), 8.22 (s, 1H).

Benzyl (*E*)-3-(2′-chloro-6-hydroxy-5-methoxy-[1,1′-biphenyl]-3-yl)-2-cyanoacrylate (**4j**).

^1^H NMR (CDCl_3_, 400 MHz): *δ* = 4.06 (s, 3H), 5.38 (s, 2H), 6.37 (s, 1H), 7.30–7.54 (m, 6H), 7.53–7.58 (m, 10H), 8.00 (s, 1H), 8.21 (s, 1H).

Benzyl (*E*)-3-(3′-chloro-6-hydroxy-5-methoxy-[1,1′-biphenyl]-3-yl)-2-cyanoacrylate (**4k**).

^1^H NMR (CDCl_3_, 400 MHz): *δ* = 4.03 (s, 3H), 5.36 (s, 2H), 6.51 (s, 1H), 7.32–7.49 (m, 9H), 7.60 (s, 1H), 7.92 (s, 1H), 8.19 (s, 1H).

Benzyl (*E*)-3-(4′-chloro-6-hydroxy-5-methoxy-[1,1′-biphenyl]-3-yl)-2-cyanoacrylate (**4l**).

^1^H NMR (CDCl_3_, 400 MHz): *δ* = 4.05 (s, 3H), 5.38 (s, 2H), 6.54 (s, 1H), 7.36–7.49 (m, 8H), 7.56 (d, *J* = 8.4 Hz, 2H), 7.89 (s, 1H), 8.22 (s, 1H).

Benzyl (*E*)-2-cyano-3-(2′-cyano-6-hydroxy-5-methoxy-[1,1′-biphenyl]-3-yl)acrylate (**4m**).

^1^H NMR (CDCl_3_, 400 MHz): *δ* = 4.05 (s, 3H), 5.40 (s, 2H), 7.36–7.49 (m, 5H), 7.67 (t, *J* = 7.4 Hz, 1H), 7.77 (s, 1H), 7.89 (t, *J* = 7.8 Hz, 1H), 8.14 (d, *J* = 8.5 Hz, 1H), 8.29 (d, *J* = 1.5 Hz, 1H), 8.31 (s, 1H), 8.44 (d, *J* = 7.8 Hz, 1H).

Benzyl (*E*)-2-cyano-3-(3′-cyano-6-hydroxy-5-methoxy-[1,1′-biphenyl]-3-yl)acrylate (**4n**).

^1^H NMR (CDCl_3_, 400 MHz): *δ* = 4.03 (s, 3H), 5.35 (s, 2H), 6.62 (s, 1H), 7.33–7.47 (m, 6H), 7.55 (d, *J* = 7.6 Hz, 1H), 7.64 (d, *J* = 7.6 Hz, 1H), 7.81 (d, *J* = 8.5 Hz, 1H), 7.89 (s, 1H), 7.93 (s, 1H), 8.19 (s, 1H).

Benzyl (*E*)-2-cyano-3-(4′-cyano-6-hydroxy-5-methoxy-[1,1′-biphenyl]-3-yl)acrylate (**4o**).

^1^H NMR (CDCl_3_, 400 MHz): *δ* = 4.02 (s, 3H), 5.35 (s, 2H), 7.34–7.47 (m, 6H), 7.72 (s, 4H), 7.86 (s, 1H), 8.19 (s, 1H).

Benzyl (*E*)-2-cyano-3-(6-hydroxy-5-methoxy-2′-(trifluoromethyl)-[1,1′-biphenyl]-3-yl)acrylate (**4p**).

^1^H NMR (CDCl_3_, 400 MHz): *δ* = 4.01 (s, 3H), 5.34 (s, 2H), 6.33 (s, 1H), 7.23 (s, 1H), 7.30–7.45 (m, 6H), 7.51 (t, *J* = 7.4 Hz, 1H), 7.59 (t, *J* = 7.4 Hz, 1H), 7.78 (d, *J* = 7.8 Hz, 1H), 7.98 (d, *J* = 1.5 Hz, 1H), 8.15 (s, 1H).

Benzyl (*E*)-2-cyano-3-(6-hydroxy-5-methoxy-3′-(trifluoromethyl)-[1, 1′-biphenyl]-3-yl)acrylate (**4q**).

^1^H NMR (CDCl_3_, 400 MHz): *δ* = 4.03 (s, 3H), 5.35 (s, 2H), 7.34–7.45 (m, 6H), 7.57 (d, *J* = 7.8 Hz, 1H), 7.62 (d, *J* = 7.8 Hz, 1H), 7.77 (d, *J* = 7.8 Hz, 1H), 7.87 (s, 1H), 7.92 (s, 1H), 8.20 (s, 1H).

Benzyl (*E*)-2-cyano-3-(6-hydroxy-5-methoxy-4′-(trifluoromethyl)-[1,1′-biphenyl]-3-yl)acrylate (**4r**).

^1^H NMR (CDCl_3_, 400 MHz): *δ* = 4.02 (s, 3H), 5.35 (s, 2H), 5.65 (s, 1H), 7.31–7.48 (m, 6H), 7.66–7.73 (m, 4H), 7.88 (s, 1H), 8.19 (s, 1H).

Benzyl (*E*)-2-cyano-3-(6-hydroxy-5-methoxy-2′-(trifluoromethoxy)-[1,1′-biphenyl]-3-yl)acrylate (**4s**).

^1^H NMR (CDCl_3_, 400 MHz): *δ* = 4.02 (s, 3H), 5.35 (s, 2H), 6.43 (s, 1H), 7.29 (s, 1H), 7.32–7.47 (m, 9H), 7.97 (s, 1H), 8.17 (s, 1H).

benzyl (*E*)-2-cyano-3-(6-hydroxy-5-methoxy-3′-(trifluoromethoxy)-[1,1′-biphenyl]-3-yl)acrylate (**4t**).

^1^H NMR (CDCl_3_, 400 MHz): *δ* = 4.02 (s, 3H), 5.35 (s, 2H), 7.22 (d, *J* = 8.0 Hz, 1H), 7.34–7.54 (m, 9H), 7.90 (s, 1H), 8.19 (s, 1H).

Benzyl (*E*)-2-cyano-3-(6-hydroxy-5-methoxy-4′-(trifluoromethoxy)-[1,1′-biphenyl]-3-yl)acrylate (**4u**).

^1^H NMR (CDCl_3_, 400 MHz): *δ* = 4.01 (s, 3H), 5.35 (s, 2H), 7.22–7.47 (m, 8H), 7.62 (d, *J* = 8.5 Hz, 2H), 7.86 (s, 1H), 8.19 (s, 1H).

Benzyl (*E*)-3-(4′-(tert-butyl)-6-hydroxy-5-methoxy-[1,1′-biphenyl]-3-yl)-2-cyanoacrylate (**4v**).

^1^H NMR (CDCl_3_, 400 MHz): *δ* = 1.36 (s, 9H), 4.00 (s, 3H), 5.35 (s, 2H), 6.52 (s, 1H), 7.33–7.55 (m, 10H), 7.86 (s, 1H), 8.19 (s, 1H).

Benzyl (*E*)-2-cyano-3-(6-hydroxy-5-methoxy-[1,1′:4′,1″-terphenyl]-3-yl)acrylate (**4w**).

^1^H NMR (CDCl_3_, 400 MHz): *δ* = 4.01 (s, 3H), 5.35 (s, 2H), 6.55 (s, 1H), 7.32–7.49 (m, 9H), 7.63 (d, *J* = 7.6 Hz, 2H), 7.67 (s, 4H), 7.88 (s, 1H), 8.20 (s, 1H).

Benzyl (*E*)-2-cyano-3-(4-hydroxy-3-methoxy-5-(thiophen-2-yl)phenyl)acrylate (**5a**).

^1^H NMR (CDCl_3_, 400 MHz): *δ* = 3.98 (s, 3H), 5.35 (s, 2H), 6.83 (s, 1H), 7.10 (t, *J* = 4.4 Hz, 1H), 7.33–7.47 (m, 6H), 7.60 (d, *J* = 3.7 Hz, 1H), 7.68 (s, 1H), 7.76 (s, 1H), 8.16 (s, 1H).

Benzyl (*E*)-2-cyano-3-(4-hydroxy-3-methoxy-5-(5-methylthiophen-2-yl)phenyl)acrylate (**5b**).

^1^H NMR (CDCl_3_, 400 MHz): *δ* = 2.51 (s, 3H), 3.98 (s, 3H), 5.35 (s, 2H), 6.76 (d, *J* = 3.3 Hz, 1H), 7.32–7.48 (m, 6H), 7.62 (s, 1H), 7.73 (s, 1H), 8.15 (s, 1H).

Benzyl (*E*)-3-(3-(5-chlorothiophen-2-yl)-4-hydroxy-5-methoxyphenyl)-2-cyanoacrylate (**5c**).

^1^H NMR (CDCl_3_, 400 MHz): *δ* = 3.99 (s, 3H), 5.36 (s, 2H), 6.92 (d, *J* = 3.9 Hz, 1H), 7.32–7.47 (m, 6H), 7.63 (s, 1H), 7.73 (s, 1H), 8.15 (s, 1H).

Benzyl (*E*)-2-cyano-3-(4-hydroxy-3-methoxy-5-(thiophen-3-yl)phenyl)acrylate (**5f**).

^1^H NMR (CDCl_3_, 400 MHz): *δ* = 3.99 (s, 3H), 5.35 (s, 2H), 6.71 (s, 1H), 7.35–7.48 (m, 6H), 7.50 (d, *J* = 5.1 Hz, 1H), 7.65 (s, 1H), 7.75–7.78 (m, 2H), 8.17 (s, 1H).

Benzyl (*E*)-2-cyano-3-(4-hydroxy-3-methoxy-5-(pyridin-4-yl)phenyl)acrylate (**6a**).

^1^H NMR (TFA salt, CDCl_3_, 400 MHz): *δ* = 4.05 (s, 3H), 5.36 (s, 2H), 7.33–7.48 (m, 5H), 7.50 (s, 1H), 7.54–7.58 (m, 2H), 7.92 (s, 1H), 8.21 (s, 1H), 8.66–8.71 (m, 2H).

Benzyl (*E*)-2-cyano-3-(4-hydroxy-3-methoxy-5-(2-methylpyridin-4-yl)phenyl)acrylate (**6b**).

^1^H NMR (TFA salt, CDCl_3_, 400 MHz): *δ* = 2.88 (s, 3H), 4.07 (s, 3H), 5.37 (s, 2H), 7.35–7.47 (m, 5H), 7.62 (s, 1H), 7.88–7.96 (m, 2H), 7.99 (d, *J* = 5.4 Hz, 1H), 8.21 (s, 1H), 8.76 (d, *J* = 5.8 Hz, 1H).

Benzyl (*E*)-2-cyano-3-(3-(2-fluoropyridin-4-yl)-4-hydroxy-5-methoxyphenyl)acrylate (**6c**).

^1^H NMR (TFA salt, CDCl_3_, 400 MHz): *δ* = 4.03 (s, 3H), 5.36 (s, 2H), 7.14 (s, 1H), 7.34–7.47 (m, 6H), 7.97 (d, *J* = 1.6 Hz, 1H), 8.18 (s, 1H), 8.48 (d, *J* = 4.9 Hz, 1H), 8.56 (s, 1H).

Benzyl (*E*)-2-cyano-3-(4-hydroxy-3-methoxy-5-(pyrimidin-5-yl)phenyl)acrylate (**6d**).

^1^H NMR (TFA salt, CDCl_3_, 400 MHz): *δ* = 4.05 (s, 3H), 5.37 (s, 2H), 6.97 (s, 1H), 7.32–7.49 (m, 7H), 7.95 (s, 1H), 8.21 (s, 1H), 9.09 (s, 2H), 9.25 (s, 1H).

Benzyl (*E*)-2-cyano-3-(4-hydroxy-3-methoxy-5-(pyridin-3-yl)phenyl)acrylate (**6e**).

^1^H NMR (TFA salt, CDCl_3_, 400 MHz): *δ* = 4.05 (s, 3H), 5.37 (s, 2H), 7.34–7.49 (m, 5H), 7.56 (s, 1H), 7.87 (s, 1H), 8.20 (s, 1H), 8.38 (s, 2H), 8.58 (s, 1H).

Benzyl (*E*)-2-cyano-3-(4-hydroxy-3-(isoquinolin-5-yl)-5-methoxyphenyl)acrylate (**6f**).

^1^H NMR (TFA salt, CDCl_3_, 400 MHz): *δ* = 4.10 (s, 3H), 5.36 (s, 2H), 6.57 (s, 1H), 7.37–7.46 (m, 6H), 7.93 (d, *J* = 6.7 Hz, 1H), 7.97–8.06 (m, 3H), 8.21 (s, 1H), 8.35 (d, *J* = 8.1 Hz, 1H), 8.50 (d, *J* = 6.5 Hz, 1H), 9.76 (s, 1H).

Benzyl (E)-2-cyano-3-(4-hydroxy-3-methoxy-5-(quinolin-5-yl)phenyl)acrylate (**6g**).

^1^H NMR (TFA salt, CDCl_3_, 400 MHz): *δ* = 4.09 (s, 3H), 5.36 (s, 2H), 7.32–7.46 (m, 6H), 7.73 (dd, *J* = 8.5, 4.9 Hz, 1H), 7.77 (d, *J* = 7.2, 4.9 Hz, 1H), 8.01 (s, 1H), 8.04 (t, *J* = 7.8 Hz, 1H), 8.21 (s, 1H), 8.49 (d, *J* = 8.5 Hz, 1H), 8.55 (d, *J* = 8.6 Hz, 1H), 9.24 (d, *J* = 4.8 Hz, 1H).

Benzyl (*E*)-2-cyano-3-(4-hydroxy-3-methoxy-5-(6-(piperazin-1-yl)pyridin-3-yl)phenyl)acrylate (**6h**).

^1^H NMR (triple TFA salt, MeOD, 400 MHz): *δ* = 3.36 (t, *J* = 5.6 Hz, 4H), 3.86 (t, *J* = 5.6 Hz, 4H), 4.00 (s, 3H), 5.37 (s, 2H), 7.15 (d, *J* = 5.6 Hz, 1H), 7.16 (s, 1H), 7.37–7.48 (m, 5H), 7.81–7.84 (m, 2H), 8.22 (d, *J* = 5.5 Hz, 1H), 8.35 (s, 1H).

2nd generation compounds **7a**-**7k**, entries 60–70, were synthesized using a combination of the 1st general procedure, to introduce different esters, and then of the 2nd general procedure to introduce the acetyl thiophene residue.

#### Analytical data of the active compounds:

Phenethyl (*E*)-3-(3-(5-acetylthiophen-2-yl)-4-hydroxy-5-methoxyphenyl)-2-cyanoacrylate (**7a**).

^1^H NMR (CDCl_3_, 400 MHz): *δ* = 8.13 (s, 1H), 7.83 (m, 1H), 7.73 (m, 1H), 7.70 (m, 1H), 7.62 (m, 1H), 7.31 (m, 5H), 4.52 (t, j = 14 Hz, 2H), 4.04 (s, 3H), 3.08 (t, *J* = 14.2 Hz, 2H), 2.59 (s, 1H). ^13^C NMR (CDCl_3_, 101 MHz): *δ* = 190.99, 162.83, 154.55, 147.58, 145.64, 143.97, 137.41, 132.76, 129.25, 128.77, 127.38, 127.35, 126.95, 124.15, 120.27, 116.21, 110.28, 100.40, 67.15, 56.79, 35.12, 26.92. HRMS (ESI neg): [M-H]^−^ calcd. For C_25_H_20_NO_5_S: 446.1068. Found: 446.1065.

4-Chlorobenzyl (*E*)-3-(3-(5-acetylthiophen-2-yl)-4-hydroxy-5-methoxyphenyl)-2-cyanoacrylate (**7b**).

^1^H NMR (CDCl_3_, 400 MHz): *δ* = 8.19 (s, 1H), 7.85 (m, 1H), 7.75 (m, 1H), 7.69 (m, 1H), 7.62 (m, 1H), 7.39 (m, 4H), 5.33 (s, 2H), 4.04 (s, 3H), 2.59 (s, 3H). ^13^C NMR (CDCl_3_, 101 MHz): *δ* = 162.75, 154.98, 147.73, 132.73, 129.86, 129.09, 127.60, 127.42, 124.11, 120.34, 116.13, 110.26, 100.15, 67.31, 56.83, 26.93. HRMS (ESI neg): [M-H]^−^ calcd. For C_24_H_17_ClNO_5_S: 466.0521. Found: 466.0524.

4-Methoxybenzyl (*E*)-3-(3-(5-acetylthiophen-2-yl)-4-hydroxy-5-methoxyphenyl)-2-cyanoacrylate (**7c**).

^1^H NMR (DMSO, 400 MHz): *δ* = 8.37 (s, 1H), 8.26 (m, 1H), 7.94 (m, 1H), 7.82 (m, 1H), 7.73 (m, 1H), 7.41 (m, 2H), 6.97 (m, 2H), 5.27 (s, 2H), 3.90 (s, 3H), 3.77 (s, 3H), 2.54 (s, 3H). ^13^C NMR (DMSO, 101 MHz): *δ* = 191.01, 162.49, 159.41, 154.86, 148.52, 145.66, 143.09, 133.40, 130.18, 127.41, 126.18, 120.35, 116.60, 113.97, 67.17, 56.18, 55.17, 26.56. HRMS (ESI neg): [M-H]^−^ calcd. For C_25_H_20_NO_6_S: 462.1017. Found: 462.1024.

2-Chlorobenzyl (*E*)-3-(3-(5-acetylthiophen-2-yl)-4-hydroxy-5-methoxyphenyl)-2-cyanoacrylate (**7d**).

^1^H NMR (CDCl_3_, 400 MHz): *δ* = 8.21 (s, 1H), 7.86 (m, 1H), 7.76 (m, 1H), 7.70 (m, 1H), 7.62 (m, 1H), 7.54 (m, 1H), 7.43 (m, 1H), 7.32 (m, 2H), 6.95 (m, 1H), 5.47 (s, 2H), 4.04 (s, 3H), 2.59 (s, 3H). ^13^C NMR (CDCl_3_, 101 MHz): *δ* = 190.94, 162.62, 154.95, 147.71, 147.60, 145.55, 144.01, 133.68, 132.91, 132.72, 129.94, 129.79, 127.53, 127.38, 127.23, 124.12, 120.32, 116.12, 110.32, 100.14, 65.44, 56.81, 26.93. HRMS (ESI neg): [M-H]^−^ calcd. For C_24_H_17_ClNO_5_S: 466.0521. Found: 466.0515.

3-Chlorobenzyl (*E*)-3-(3-(5-acetylthiophen-2-yl)-4-hydroxy-5-methoxyphenyl)-2-cyanoacrylate (**7e**).

^1^H NMR (CDCl_3_, 400 MHz): *δ* = 8.20 (s, 1H), 7.85 (m, 1H), 7.75 (m, 1H), 7.70 (m, 1H), 7.62 (m, 1H), 7.44 (s, 1H), 7.34 (m, 3H), 6.93 (s, 1H), 5.33 (s, 2H), 4.04 (s, 3H), 2.59 (s, 3H). ^13^C NMR (CDCl_3_, 101 MHz): *δ* = 90.92, 162.71, 155.06, 147.75, 145.51, 144.04, 137.13, 134.76, 132.71, 130.19, 128.94, 128.46, 127.60, 127.40, 126.45, 124.09, 120.34, 116.11, 110.29, 100.06, 67.18, 56.82, 26.93. HRMS (ESI neg): [M-H]^−^ calcd. For C_24_H_17_ClNO_5_S: 466.0521. Found: 466.0515.

2-Methoxybenzyl (*E*)-3-(3-(5-acetylthiophen-2-yl)-4-hydroxy-5-methoxyphenyl)-2-cyanoacrylate (**7f**).

^1^H NMR (CDCl_3_, 400 MHz): *δ* = 8.19 (s, 1H), 7.85 (m, 1H), 7.74 (m, 1H), 7.70 (m, 1H), 7.61 (m, 1H), 7.44 (m, 1H), 7.35 (m, 1H), 6.96 (m, 3H), 5.42 (s, 2H), 4.03 (s, 3H), 3.88 (s, 3H), 2.59 (s, 3H). ^13^C NMR (CDCl_3_, 101 MHz): *δ* = 190.95, 162.86, 157.66, 155.76, 154.46, 147.57, 145.66, 143.95, 132.73, 130.01, 129.67, 127.34, 127.32, 124.20, 123.57, 120.70, 120.25, 116.25, 113.18, 110.71, 110.31, 100.73, 63.84, 56.80, 55.67, 26.92. HRMS (ESI neg): [M-H]^−^ calcd. For C_25_H_20_NO_6_S: 462.1017. Found: 462.1013.

3-Methoxybenzyl (*E*)-3-(3-(5-acetylthiophen-2-yl)-4-hydroxy-5-methoxyphenyl)-2-cyanoacrylate (**7g**).

^1^H NMR (CDCl_3_, 400 MHz): *δ* = 8.17 (s, 1H), 7.82 (m, 1H), 7.73 (m, 1H), 7.68 (m, 1H), 7.60 (m, 1H), 7.31 (m, 1H), 7.02 (m, 3H), 6.90 (m, 1H), 5.33 (s, 2H), 4.02 (s, 3H), 3.83 (s, 3H), 2.58 (s, 3H). ^13^C NMR (CDCl_3_, 101 MHz): *δ* = 190.99, 162.76, 159.96, 154.74, 147.66, 145.60, 143.94, 136.66, 132.74, 129.90, 127.33, 124.09, 120.48, 120.26, 116.17, 114.31, 113.68, 110.29, 100.30, 67.94, 56.77, 55.44, 26.90. HRMS (ESI pos): [M + H]^+^ calcd. For C_25_H_22_NO_6_S: 464.1162. Found: 464.1168.

2-Methylbenzyl (*E*)-3-(3-(5-acetylthiophen-2-yl)-4-hydroxy-5-methoxyphenyl)-2-cyanoacrylate (**7h**) (**PRLthiophenib**).

^1^H NMR (CDCl_3_, 400 MHz): *δ* = 8.18 (s, 1H), 7.84 (m, 1H), 7.74 (m, 1H), 7.70 (m, 1H), 7.61 (m, 1H), 7.43 (m, 1H), 7.25 (m, 3H), 6.91 (s, 1H), 5.38 (s, 2H), 4.04 (s, 3H), 2.59 (s, 3H), 2.44 (s, 3H). ^13^C NMR (CDCl_3_, 101 MHz): *δ* = 190.93, 162.77, 154.66, 147.61, 145.59, 144.00, 137.37, 133.15, 132.72, 130.68, 129.53, 129.05, 127.42, 127.37, 126.27, 124.16, 120.30, 116.17, 110.29, 100.46, 66.73, 56.81, 26.93, 19.18. HRMS (ESI neg): [M-H]^−^ calcd. For C_25_H_20_NO_5_S: 446.1068. Found: 446.1065.

3-Methylbenzyl (*E*)-3-(3-(5-acetylthiophen-2-yl)-4-hydroxy-5-methoxyphenyl)-2-cyanoacrylate (**7i**).

^1^H NMR (CDCl_3_, 400 MHz): *δ* = 8.19 (s, 1H), 7.85 (m, 1H), 7.74 (m, 1H), 7.70 (m, 1H), 7.61 (m, 1H), 7.28 (m, 3H), 7.18 (m, 1H), 6.91 (s, 1H), 5.33 (s, 2H), 4.04 (s, 3H), 2.59 (s, 3H), 2.39 (s, 3H). ^13^C NMR (CDCl_3_, 101 MHz): *δ* = 190.92, 162.83, 154.69, 147.59, 145.59, 144.01, 135.11, 132.71, 129.52, 129.22, 128.76, 127.45, 127.37, 125.57, 124.17, 120.30, 116.20, 110.28, 100.52, 68.24, 56.81, 26.93, 21.54. HRMS (ESI neg): [M-H]^−^ calcd. For C_25_H_20_NO_5_S: 446.1068. Found: 446.1070.

4-Methylbenzyl (*E*)-3-(3-(5-acetylthiophen-2-yl)-4-hydroxy-5-methoxyphenyl)-2-cyanoacrylate (**7j**).

^1^H NMR (CDCl_3_, 400 MHz): *δ* = 8.17 (s, 1H), 7.84 (m, 1H), 7.73 (m, 1H), 7.70 (m, 1H), 7.61 (m, 1H), 7.35 (m, 2H), 7.21 (m, 2H), 6.91 (s, 1H), 5.32 (s, 2H), 4.03 (s, 3H), 2.59 (s, 3H), 2.37 (s, 3H). ^13^C NMR (CDCl_3_, 101 MHz): *δ* = 190.93, 162.84, 154.61, 147.58, 145.61, 132.20, 129.52, 128.68, 127.42, 127.37, 124.17, 120.28, 116.19, 110.26, 100.58, 68.18, 56.81, 26.93, 21.39. HRMS (ESI pos): [M + H]^+^ calcd. For C_25_H_22_O_5_NS: 448.1213. Found: 448.1216. Calcd. for C_25_H_21_O_5_NNaS: 470.1033. Found: 470.1036.

Thiophen-3-ylmethyl (*E*)-3-(3-(5-acetylthiophen-2-yl)-4-hydroxy-5-methoxyphenyl)-2-cyanoacrylate (**7k**).

^1^H NMR (CDCl_3_, 400 MHz): *δ* = 8.18 (s, 1H), 7.85 (m, 1H), 7.74 (m, 1H), 7.70 (m, 1H), 7.61 (m, 1H), 7.42 (m, 1H), 7.35 (m, 1H), 7.81 (m, 1H), 6.92 (s, 1H), 5.37 (s, 2H), 4.04 (s, 3H), 2.59 (s, 3H). ^13^C NMR (CDCl_3_, 101 MHz): *δ* = 190.97, 162.78, 154.77, 147.62, 147.57, 145.57, 143.98, 135.94, 132.75, 127.75, 127.48, 127.37, 126.62, 125.20, 124.11, 120.27, 116.19, 110.22, 100.34, 63.21, 56.80, 26.93. HRMS (ESI pos): [M + H]^+^ calcd. For C_22_H_18_O_5_NS_2_: 440.0621. Found: 440.0624.

### 4.2. Usage of JMS-053

JMS-053 (HY-135457, MedChemExpress) stock solutions (10 mM in DMSO, anhydrous, ≥99.9 %, 276855 Sigma Aldrich) were prepared according to manual. After the JMS-053 powder was dissolved in DMSO, it was stored at −20 °C and used no longer than 2 weeks.

### 4.3. In vitro phosphatase activity and residual phosphatase activity assay

All phosphatase activity and inhibition assays were carried out using DiFMUP (6,8-difluoro-4-methylumbelliferyl phosphate; Invitrogen) as artificial substrate and in OptiPlate - 96 F black 96-well plates (PerkinElmer). Recombinant enzymes were prepared as described previously by Hoeger et al.^24^ Recombinant PRL-3 was used at 35 nM concentration for 1st generation compounds and at 50 nM for second generation in phosphatase activity assay buffer (20 mM Tris-Cl, pH 7.5, 150 mM NaCl, 10 mM DTT, 0.01 % Triton-X 100, 5 % DMSO) and was at least 15 min incubated with buffer prior the assay to ensure full activity due to the reducing agent dithiothreitol (DTT) included in the buffer. Enzyme reaction kinetics were monitored at 358 nm excitation/ 452 nm emission for 20 min on a Synergy H4 Hybrid multiwell plate reader (BioTek) after addition of the substrate DiFMUP (21 μM). Inhibition kinetics were obtained using inhibitor concentration series (1:2 dilutions). Control dilution series without enzyme were used to subtract the inhibitor baseline from the data. IC_50_ values for PRL-3 were calculated by plotting obtained initial velocities *versus* inhibitor concentration (logarithmic scale) using *GraphPad Prism 6* software: Nonlinear regression: log(inhibitor) *vs.* response – Variable slope (four parameters). All PRL-3 IC_50_ values were obtained from triplicates in at least three independent experiments.

Residual phosphatase activity assays were carried out in a similar way except that the inhibitor concentration was set to 30 μM for all experiments and DiFMUP substrate concentration was set dependent on the *K*_*m*_ of the enzymes (21 μM for PRL-3; 24 μM for PRL-1, PRL-2, PTP1B and TCPTP; 90 μM for VHR).^21^ For the enzymes PTP1B and TCPTP, BSA containing assay buffer (19 mM HEPES, pH 7.2, 300 mM NaCl, 0.1 mM DTT, 1.5 mM EDTA, 0.5 % Glycerol, 0.01 % BSA (ultrapure), 0.0012 % Brij-96, 5 % DMSO)^37^ was used. To ensure similar activity PRL-3 was used at 50 nM concentration, PRL-1 at 150 nM and PRL-2 at 100 nM (see Supporting Fig. S2). Due to higher activity, the concentration of PTP1B, TCPTP, and VHR was set to 0.5 nM.^24^ The initial velocities of each enzyme with inhibitor were normalized to the respective control without inhibitor to obtain the residual phosphatase activity. Assays were performed in triplicates in at least two independent experiments.

### 4.4. Short- / long-term cytotoxicity

Doxycycline (dox)-inducible PRL-3 expressing HEK293 stable cell lines were prepared and cultured as previously described.^24^ For short-term cytotoxicity experiments, dox-induced (1 μg/mL; Sigma) HEK293 stable cell lines were seeded into a sterile 96-well transparent plate (Greiner) with a density of 10,000 cells/well. After 24 h at 37 °C, compounds or DMSO control were added at the indicated concentrations. After 16 h incubation at 37 °C, the medium was removed, and fresh phenol red-free growth medium was added containing 3-(4,5-dimethylthiazol-2-yl)-2,5-diphenyltetrazolium bromide (MTT, 5 mg/mL stock in PBS, used 1:10 in growth medium; Sigma) and for 4 h incubated at 37 °C. The medium was then carefully removed and the living cells, which formed formazan crystals were dissolved in DMSO (100 μL/well) by thorough pipetting. After 10 min incubation at room temperature, absorbance was determined at 540 nm on a Synergy H1 multiwell plate reader (Biotek) and the phenol red baseline at 690 nm was subtracted.

Long-term cytotoxicity experiments were carried out it in a similar way. The cells were seeded in a density of 1500 cells/well and treated with respective concentration of compounds or DMSO control for 9 days. Every 3 days growth medium containing dox and respective compound concentration was changed. At the indicated days the respective wells were treated with MTT and analyzed as described.

### 4.5. Fluorescence polarization assay for binding

The autofluorescence of PRLthiophenib (**7h**) allowed to perform binding experiments using fluorescence anisotropy. Fluorescence polarization (FP) assays were conducted to measure the binding interactions between the autofluorescent ligand PRLthiophenib (**7h**) and the target protein PRL-3. The assays were performed in black, flat-bottom 384-well microplates (Corning, #3575) using a total reaction volume of 40 μL per well. Increasing concentrations of the target protein (0.15 to 300 μM) were incubated with a fixed concentration of PRLthiophenib (10 μM) in phosphatase assay buffer for 30 min at room temperature. Fluorescence polarization measurements were taken using a Synergy H1 plate reader (BioTek) with a blue filter set (excitation: 485/20 nm, emission: 528/20 nm) and fluorescence endpoint measurement. The FP values were plotted against the logarithm of protein concentration, and the dissociation constant (*K*_d_) was determined by fitting the data to a one-site binding model using GraphPad Prism software (version 6.0).

### 4.6. Determination of the mode of inhbition of PRLthiophinib (7h)

The mode of inhibition of PRLthiophinib (**7h**) was determined using the DiFMUP assay (see above) with 50 nM of recombinant PRL-3 and varying substrate (DiFMUP) concentrations. Enzymatic activity was measured in the absence and presence of increasing concentrations of the inhibitor.

For kinetic analysis, a Lineweaver–Burk plot was generated using linear regression to assess the type of inhibition.^24^ In addition, a Michaelis–Menten curve was fitted using nonlinear regression (Michaelis–Menten model) in GraphPad Prism software (version 6.0).

### 4.7. Computational methods

Preparation of Protein and Ligand: The NMR-derived structure of phosphatase of regenerating liver-3 (PRL-3) was obtained from the RCSB Protein Data Bank^32^ (RCSB.org) under accession code 1V3A, which includes a single conformer. Following download, the 1V3A structure was submitted to CB-DOCK2^33^ (http://cao.labshare.cn/cb-dock2/), an automated online platform that processes protein structures by removing solvent molecules and non-standard ligands, adding missing hydrogen atoms, assigning protonation states at physiological pH, and converting the final coordinates into PDBQT format. The small-molecule ligand, PRLthiophenib, was uploaded to CB-DOCK2 in Structure Data File (SDF) format. CB-DOCK2 then processed the ligand by adding missing hydrogen atoms, assigning partial atomic charges, adjusting protonation states (~pH 7.4), and converting it to a PDBQT file compatible with AutoDock Vina.^34^

Blind Docking and Binding Site Prediction: Following protein and ligand preparation, CB-DOCK2 conducted blind docking by predicting up to five potential binding sites on the PRL-3 surface. For each predicted binding site, the software generated a search grid (center coordinates and box dimensions) and utilized AutoDock Vina to determine possible binding modes. The outputs included: (1) the best docking pose, (2) the corresponding Vina score (kcal/mol), (3) the cavity volume (Å^3^), and (4) the spatial coordinates of each identified pocket.

Visualization and Analysis of Docked Complex: Docking results were compiled into a ranked list based on the Vina score, where more negative values indicated stronger predicted binding affinity. The best pose for PRLthiophenib in the highest-ranking cavity (C1) was visualized using ChimeraX^35^ (https://www.rbvi.ucsf.edu/chimerax) for 3D representation of the docked complex and BIOVIA Discovery Studio Visualizer (https://discover.3ds.com/discovery-studio-visualizer-download) for 2D interaction mapping. In Discovery Studio Visualizer, a schematic was generated to illustrate hydrogen bonds, alkyl interactions, and other potential contacts between PRLthiophenib and PRL-3 residues.

Molecular Dynamics Simulations: To explore the stability of the top-ranked docked complex in C1, a 100 ns molecular dynamics (MD) simulation was conducted using GROMACS^38^ 2024.4 (http://www.gromacs.org) with the CHARMM36 force field, optimized for protein-ligand systems. The highest-affinity pose from the docking results was solvated in a dodecahedral box with TIP3P water molecules, ensuring at least a 1.0 nm buffer between any protein atom and the box edge. Neutralizing counter ions (Na^+^ and Cl^−^) were added to maintain overall charge balance. Energy minimization was performed using the steepest descent algorithm for up to 50,000 steps until convergence criteria were met. This was followed by short NVT (100 ps) and NPT (100 ps) equilibration phases at 300 K and 1 bar. The NVT phase used the v-rescale thermostat, while the NPT phase employed both the v-rescale thermostat and the C-rescale barostat for enhanced stability. A 100 ns production MD simulation was conducted under NPT conditions, using a 2 fs time step and periodic boundary conditions. Long-range electrostatics were treated *via* the Particle Mesh Ewald (PME) method, and all bonds involving hydrogen atoms were constrained with the LINCS algorithm. Trajectory snapshots were recorded every 10 ps for post-simulation analysis.

Post-Simulation Analysis: Throughout the simulation, the Root Mean Square Deviation (RMSD) of both the protein and ligand was monitored to assess overall stability and identify any major conformational changes. Per-residue Root Mean Square Fluctuation (RMSF) analyses were conducted to pinpoint flexible protein regions that might facilitate binding. Additionally, the radius of gyration (Rg) was calculated over the course of the simulation to characterize changes in protein compactness. Python’s Matplotlib library was used to visualize the results.

### 4.8. Intestinal organoid methods

HA-PRL-3/R26-rtTA mice were maintained on a C57Bl/6N background. Mice were kept under specific pathogen-free (SPF) conditions in the animal facility at Center for Experimental Models and Transgenic Service Freiburg according to institutional guidelines. Animals received standard diet and water *ad libitum*. Small intestine (SI) was isolated from sacrificed mice in accordance with the German law for animal protection and was approved by the government commission for animal protection and the local ethics committee (X-17/06C; X-22/05D). For SI organoid preparation heterozygous mice for the human PRL-3 gene *PTP4A3* and the reverse tetracycline-controlled transactivator (rtTA) controlled by the *Rosa26* promotor were used. Organoids were generated and cultured as described previously.^6^

Briefly, the proximal small intestine of one mouse per organoid culture was extracted, washed with cold PBS, opened, and villi were removed using a glass slide. Tissues were then cut into 3–5 mm segments. These segments were vortexed in PBS until clean, then treated with cold EDTA (2 mM in PBS) for 1 h at 4 °C on a rotator. Post-EDTA, crypts were detached by shaking in PBS with BSA (0.1 %) and filtered through a cell strainer (70 μm). After up to ten repetitions, crypts were collected, washed, and embedded in Matrigel for SI organoid cultivation in a 24-well plate, followed by the addition of IntestiCult.

For inhibitor assessment around 50 crypts of SI organoids in 10 μl diluted Matrigel (Growth Factor Reduced Basement Membrane Matrix, phenol red-free; Corning cat. no. 356238; diluted with PBS to 3 mg/mL protein concentration) were seeded per well in a sterile 96-well transparent plate (Greiner). After Matrigel polymerization at 37 °C, 100 μl per well of IntestiCult Organoid Growth Medium (Mouse; Stemcell technologies cat. no. 06005) was added. Organoids were grown for 3 days at 37 °C and 5 % CO_2_ in a humified incubator until the crypt-villi structure has formed and then induced with dox (0.3 μg/mL) and treated with the respective concentration of compound or DMSO vehicle by media change in at least triplicates. After 24 h incubation at 37 °C images of organoid phenotype were obtained by Eclipse Ts2R microscope (Plan Fluor 4×/0.13 ND, 16.5 WD, cover slide thickness 1.2; Nikon).

### 4.9. SI organoid counting and quantification

For organoid counting *CellCounter* plugin in *ImageJ* software was used. General counting criteria: organoids with a bigger diameter than 20 μm, organoids on the edge of image just if more than 50 % is visible; not counted: organoids out of focus, spheroids (circles with thin transparent cell layer). Organoids were further categorized into alive and dying. Alive organoids were defined as organoids containing sharp edges and displaying bright contrast. Very big organoids showing black centers are still considered alive when having a lot of branches and a closed outline. Dying organoids are considered, if more than 50 % of the organoid edge is dissolved, displaying dark contrast or having no branches at all (but with dark center in contrast to spheroids).

For quantification 50 to 200 organoids per condition were counted. Percentage alive organoids of total counted organoids for each well was calculated and normalized to DMSO vehicle without dox.

### 4.10. Compound stability assay in HeLa cell lysate

Compound stability was assessed in HeLa cell lysate. Compounds (10 mM in DMSO, anhydrous, ≥99.9 %, 276855–100mL, Sigma Aldrich) were diluted to 100 μM in HeLa lysate (0.8 mg/mL protein in PBS) and incubated at 37 °C in black 96-well plates (Costar, 3603). At predetermined time points, 10 μl aliquots were transferred to 190 μl of 1:1 water:methanol mixture in an Agilent plate (5043–9312), sealed with Nacalai USA Inc. RAPID Slit Seal (NC1831702) and analyzed on the Agilent RapidFire 365 High-Throughput Mass Spectrometry System. For analysis of the stability, we developed a solid-phase extraction mass spectrometry (SPE-MS/MS) assay using an Agilent RapidFire 365 instrument, equipped with an Agilent 6495 triple quadrupole mass spectrometer. For RapidFire sample processing, a phenyl SPE cartridge (Agilent G9208A) was used for sample cleanup. Cartridges were washed with 50 mM aq. ammonium formate and eluted with 0.1 % formic acid in MeOH onto the electrospray MS, where the mass spectra were collected in positive mode (PRLthiophenib, JMS-053) or negative mode (Analog 3). The RapidFire sipper was washed between sample injections using water and CH3CN. Data was processed and analyzed using Agilent MassHunter Software (RapidFire Integrator).

## Supplementary Material

1

## Figures and Tables

**Table 1:** Results of the structure-activity relationship study of 1st and 2nd generation synthesized analogs of Analog 3. Compounds were tested at 100 μM concentration for inhibiting PRL-3 in a DiFMUP dephosphorylation assay. Considerably active analogs were evaluated for their IC_50_s ± SEM. Analogs with an IC_50_ greater than 100 μM were regarded inactive. Experiments were carried out at least three times in triplicates.

Entry	Structure	Compound	R1	R2	R3	IC_50_ (μM) PRL-3

	1st Generation					
1	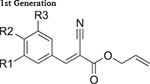	1a	H	OH	H	inactive
2		1b	OMe	OH	Cl	inactive
3		1c	OMe	OH	F	78.9 ± 27.3
4		1d	OMe	OH	Me	inactive
5		1e	OH	OMe	Br	inactive
6		1f	OMe	OMe	Br	inactive
7		1g	OMe	H	Br	inactive

8	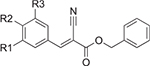	2a	OMe	OH	Br	35.5 ± 5.8
9		2b	H	OH	H	inactive
10		2c	OMe	OH	Cl	47.5 ± 1.5
11		2d	OMe	OH	F	81.9 ± 5.9
12		2e	OMe	OH	Me	inactive
13		2f	OH	OMe	Br	inactive
14		2g	OMe	OMe	Br	inactive
15		2h	OMe	H	Br	inactive

16	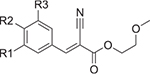	3a	OMe	OH	Br	92.2 ± 10.5
17		3b	H	OH	H	inactive
18		3c	OMe	OH	Cl	inactive
19		3d	OMe	OH	F	inactive
20		3e	OH	OMe	Br	inactive
21		3f	OMe	OMe	Br	inactive
22		3g	OMe	H	Br	inactive

23	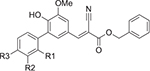	4a	Me	H	H	inactive
24		4b	H	Me	H	inactive
25		4c	H	H	Me	inactive
26		4d	F	H	H	inactive
27		4e	H	F	H	inactive
28		4f	H	H	F	inactive
29		4g	OMe	H	H	inactive
30		4h	H	OMe	H	inactive
31		4i	H	H	OMe	inactive
32		4j	Cl	H	H	inactive
33		4k	H	Cl	H	inactive
34		4l	H	H	Cl	inactive
35		4m	CN	H	H	inactive
36		4n	H	CN	H	inactive
37		4o	H	H	CN	inactive
38		4p	CF_3_	H	H	inactive
39		4q	H	CF_3_	H	inactive
40		4r	H	H	CF_3_	inactive
41		4s	OCF_3_	H	H	inactive
42		4t	H	OCF_3_	H	inactive
43		4u	H	H	OCF_3_	inactive
44		4v	H	H	tBu	inactive
45		4w	H	H	phenyl	inactive

46	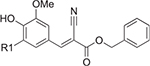	5a	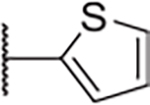			inactive
47		5b	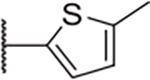			inactive
48		5c	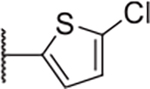			inactive
49		5d	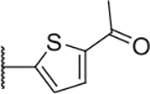			18.7 ± 2.1
50		5e	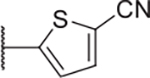			14.2 ± 2.3
51		5f	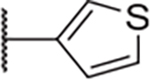			inactive

52	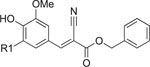	6a	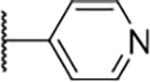			inactive
53		6b	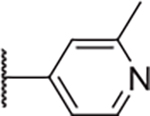			inactive
54		6c	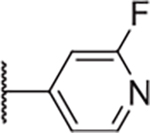			inactive
55		6d	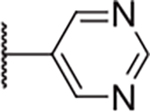			inactive
56		6e	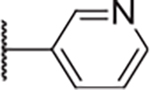			inactive
57		6f	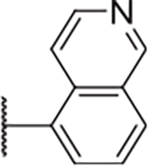			inactive
58		6g	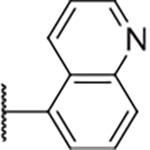			inactive
59		6h	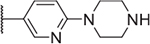			inactive

	2nd Generation					

60	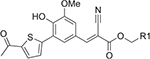	7a	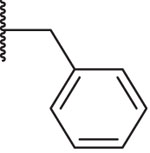			22.7 ± 4.3
61		7b	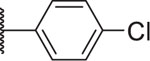			6.05 ± 0.8
62		7c	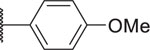			34.8 ± 5.8
63		7d	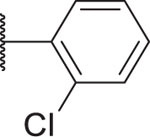			16.8 ± 2.3
64		7e	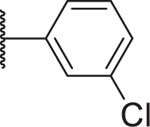			23.4 ± 0.9
65		7f	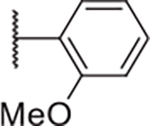			6.5 ± 1.1
66		7g	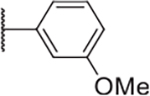			27.4 ± 1.6
67		7h PRLthiophenib	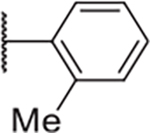			7.8 ± 1.3 (PRL-3) 31.4 ± 2.3 (PRL-2) 15.7 ± 2.2 (PRL-1)
68		7i	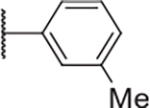			14.3 ± 7.9
69		7j	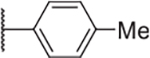			10.9 ± 0.7
70		7k	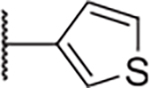			9.2 ± 0.3

**Table 2:** Phosphatase selectivity study with Analog 3 and the hit compounds. Results are shown as percentage ± SEM of residual *in vitro* phosphatase activity after inhibition at 30 μM compound concentration. DiFMUP substrate concentration was used at the *K_m_* value of the respective enzyme as well as buffer conditions. Enzyme concentration was adjusted to reach similar enzyme activity. Experiments were carried out at least two times in triplicates.

Compound	PRL-3	PRL-2	PRL-1	VHR	TCPTP	PTP1B

(30 μM)	(50 nM)	(100 nM)	(150 nM)	(0.5 nM)		
Analog 3	45 % ± 10 %	72 % ± 2 %	73 % ± 2 %	88 % ± 2 %	72 % ± 2 %	72 % ± 6 %
7b	Full inhibition	28 % ± 8 %	12 % ± 2 %	42 % ± 7 %	94 % ± 4 %	53 % ± 3 %
7f	10 % ± 5 %	39 % ± 11 %	38 % ± 4 %	75 % ± 5 %	95 % ± 5 %	57 % ± 11 %
7 h	Full inhibition	69 % ± 4 %	43 % ± 0.2 %	87 % ± 7 %	80 % ± 3 %	72 % ± 5 %
7 k	10 % ± 8 %	60 % ± 6 %	51 % ± 2 %	87 % ± 2 %	69 % ± 2 %	50 % ± 10 %

## Data Availability

Data will be made available on request.

## Appendix A. Supplementary data

Supplementary data to this article can be found online at https://doi.org/10.1016/j.bmc.2025.118412.

## Abbreviations:

DiFMUP: 6,8-difluoro-4-methylumbelliferyl phosphate
DMSO: dimethyl sulfoxide
Dox: doxycycline
HA: hemagglutinin
ISC: intestinal stem cell
MTT: 3-(4,5-dimethylthiazol-2-yl)-2,5-diphenyltetrazolium bromide
PRL: phosphatase of regenerating liver
rtTA: tetracycline-controlled transactivator
TCPTP: (T cell) protein tyrosine phosphatase
USR: ultrafast shape recognition
VHR: *Vaccinia* H1-related
