# Supplementary material for: Discovery of inhibitors of the cancer-promoting phosphatase PRL-3 and their evaluation in intestinal organoids
Source: Bioorg Med Chem. Author manuscript; Available in PMC 2026 Aug 4. (PMC13433869; doi:10.1016/j.bmc.2025.118412)
Supplement: 1 [file NIHMS2183320-supplement-1.pdf]

Supporting Information

Discovery of inhibitors of the cancer-promoting phosphatase PRL-3 and their evaluation in intestinal organoids

Andreas Hoffmann[a,b], Judith Weyershaeuser[a,b], Yamini Chand[a,b,c], Raphael Geißen[a,d], Nico Höfflin[a,b], Birgit Hoeger[e], Maja Köhn[a,b,c,e,*]

[a] Faculty of Biology, University of Freiburg, 79104 Freiburg, Germany

[b] Signalling Research Centres BIOSS and CIBSS, University of Freiburg, 79104 Freiburg, Germany

[c] Institute for Cell Biology, University of Bonn, 53115 Bonn, Germany

[d] Chemical Biology Program, Sloan Kettering Institute, Memorial Sloan Kettering Cancer Center, New York, NY 10065, United States

[e] Genome Biology Unit, EMBL Heidelberg, 69117 Heidelberg, Germany

* Correspondence: mkoehn@uni-bonn.de

Table of contents

Supporting Figure S1. Cytotoxicity of compound 5d and 5e……………………………………………………………S2

Supporting Figure S2. Titration of human recombinant PRLs to find suitable enzyme concentration with similar phosphatase activity for residual phosphatase activity assays………………………………………………………..S2

Supporting Figure S3. Sigmoidal curves for inhibition of hit compounds against the PRLs……………………..S3

Supporting Table 1. Summary of CB-DOCK2 Blind Docking Results for *PRLthiophenib* with PRL-3……………..S4

Supporting Figure S4. Computational docking of *PRLthiophenib* to the structure of PRL-3………………………S5

Supporting Figure S5. Molecular Dynamics Simulations of *PRLthiophenib* binding to PRL-3……………………S6

Supporting Figure S6. Sigmoidal curve for inhibition of JMS-053 against PRL-3……………………………………S6

Supporting Figure S7. JMS-053 does not rescue the apoptotic effect induced by PRL-3 expression in SI organoids………...S7

Supporting Figure S8. 1H-NMR and 13C-NMR spectra of Analog 3 and hit compounds……………...................S8

Supporting Figure S9. HPLC-MS spectra of Analog 3 and hit compounds…………………………………………S13

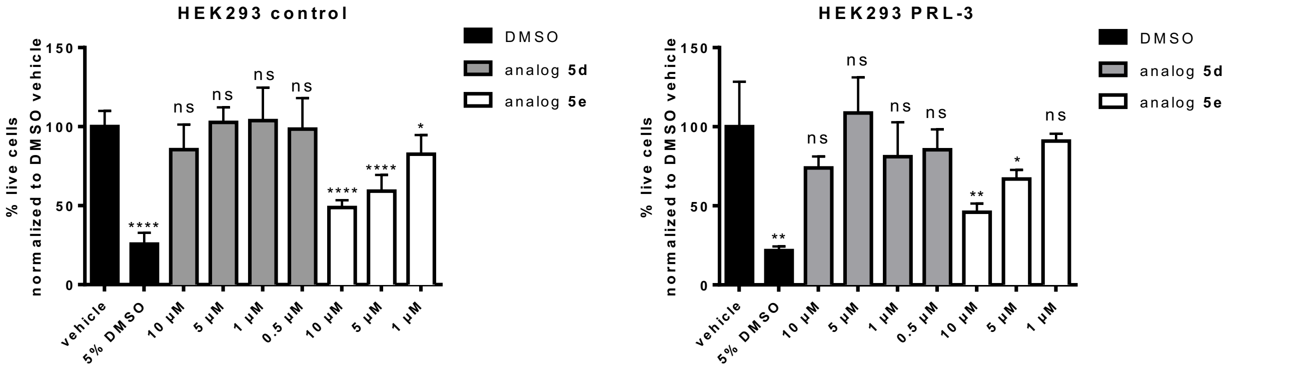

Supporting Figure S1. Cytotoxicity of compound 5d and 5e. Indicated HEK293 cells were treated for 16 h with the respective compound at different concentrations. Afterwards viability was determined using the MTT assay. Experiments were carried out in two independent experiments in triplicates, unpaired t-test was performed for $p < 0.05$, ns: not significant. Results are depicted as mean + SD.

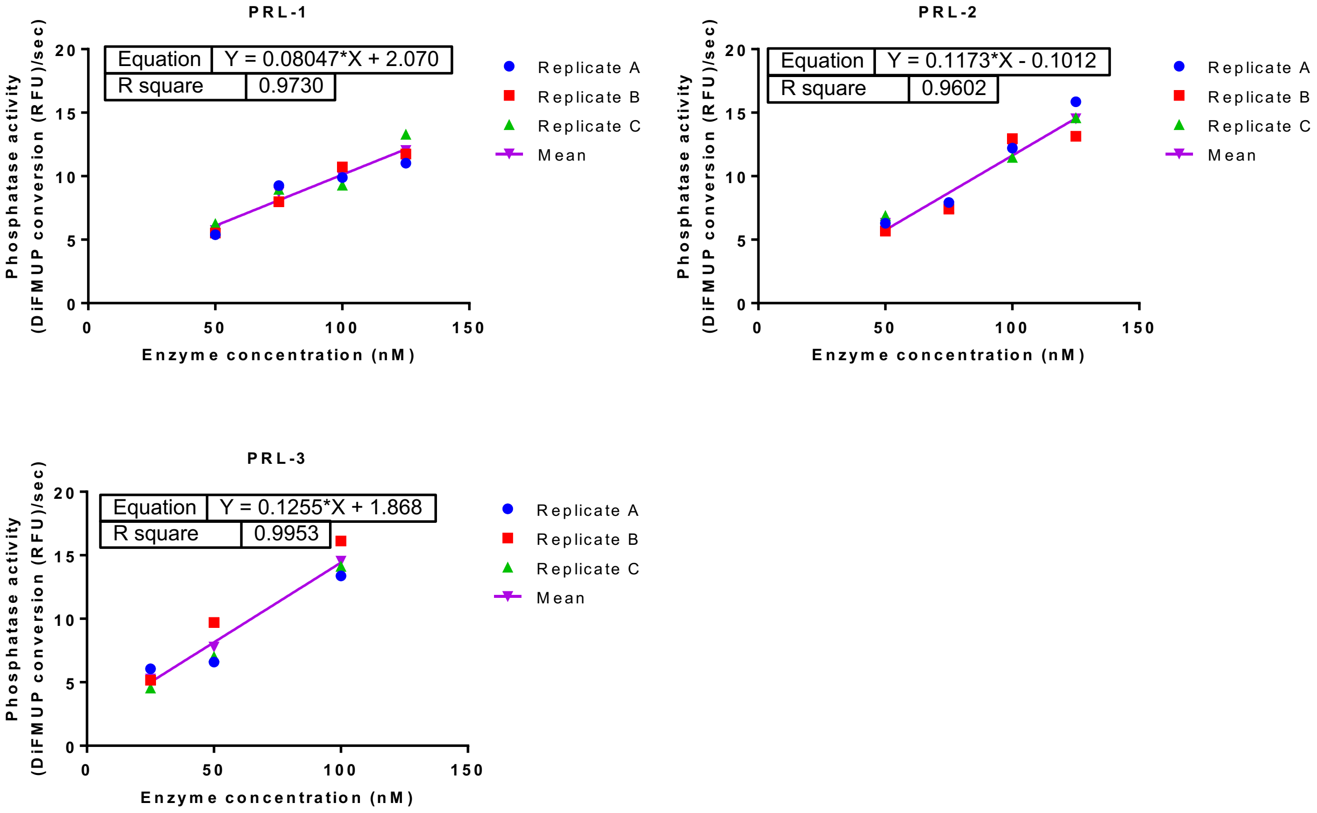

Supporting Figure S2. Titration of human recombinant PRLs to find suitable enzyme concentration with similar phosphatase activity for residual phosphatase activity assays. The DiFMUP assay was used. Experiment was carried out once in three technical replicates (data points).

a)

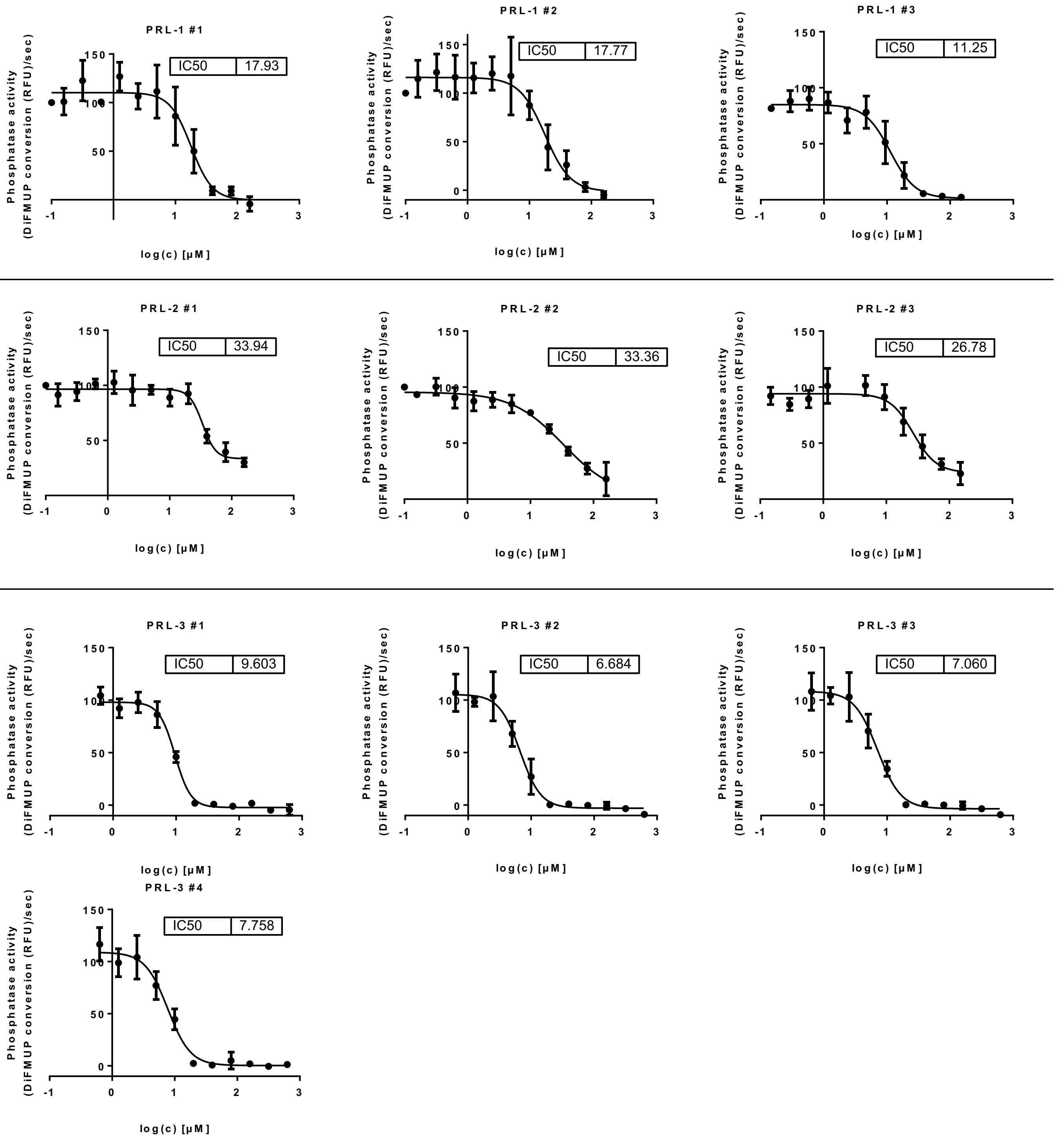
PRL-1 #1
IC50 17.93
PRL-1 #2
IC50 17.77
PRL-1 #3
IC50 11.25
PRL-2 #1
IC50 33.94
PRL-2 #2
IC50 33.36
PRL-2 #3
IC50 26.78
PRL-3 #1
IC50 9.603
PRL-3 #2
IC50 6.684
PRL-3 #3
IC50 7.060
PRL-3 #4
IC50 7.758
Phosphatase activity
(DiFMUP conversion (RFU)/sec)
log(c) [µM]

b)

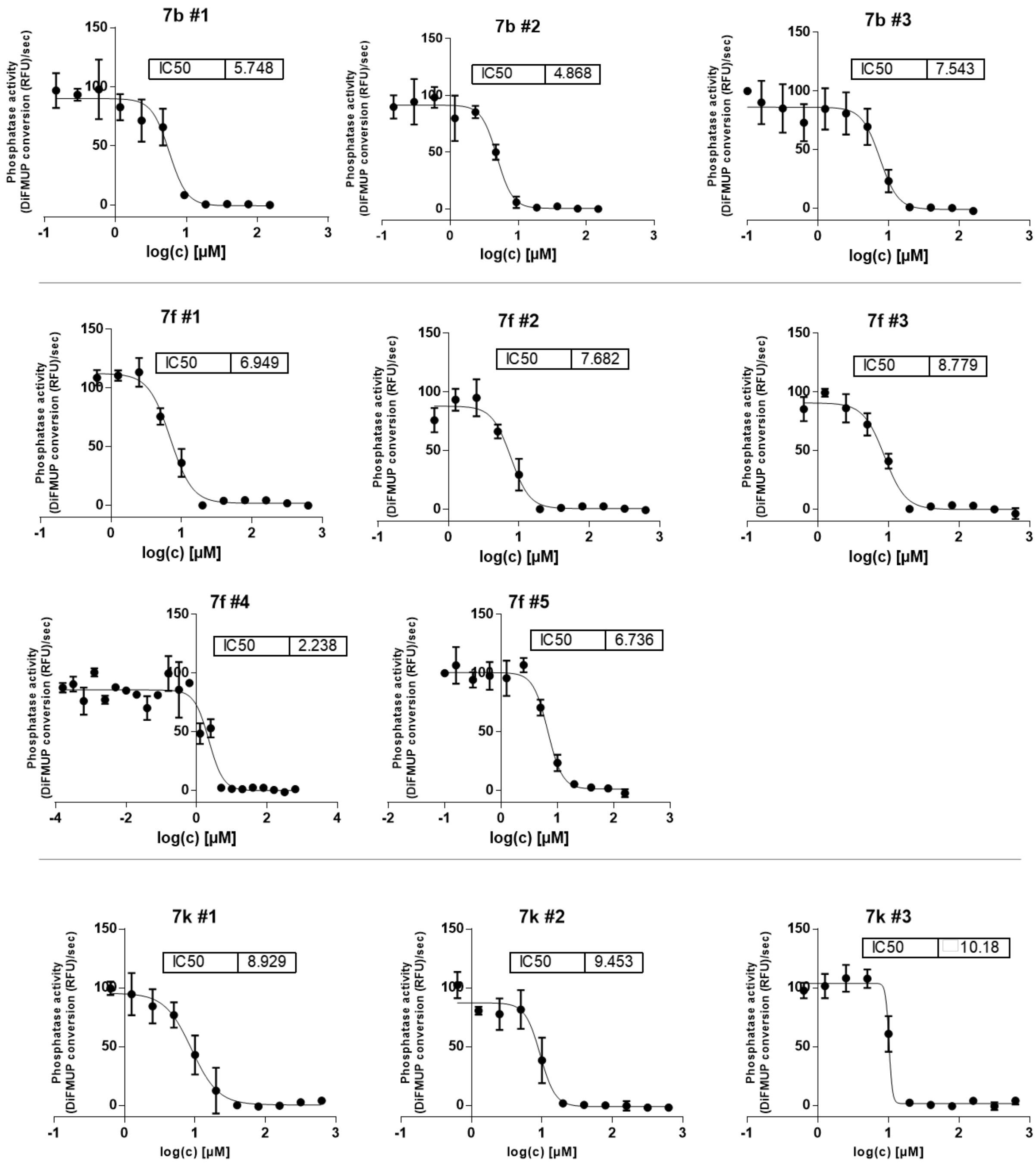

Supporting Figure S3. Sigmoidal curves for inhibition of *PRLthiophenib* (7h) against the PRLs (a) and for hit compounds 7b, 7f, and 7k against PRL-3 (b). Experiments were carried out in at least three independent experiments in triplicates. Results are depicted as mean ± SD. IC_{50}s were calculated using GraphPad Prism6 software, Nonlinear regression: log(inhibitor) vs. response -- Variable slope (four parameters).

Supporting Table 1. Summary of CB-DOCK2 Blind Docking Results for *PRLthiophenib* with PRL-3 (PDB: 1V3A). CB-DOCK2 identified five potential binding sites on PRL-3 (C1–C5). Each site is characterized by its Vina score (kcal/mol), cavity volume (Å³), and grid center coordinates (x, y, z). A more negative Vina score indicates stronger predicted binding affinity. Among these, C1 emerged as the top-scoring cavity, suggesting it as the primary binding site for *PRLthiophenib* (compound **7h**).

**Pocket ID**	**Vina Score (kcal/mol)**	**Cavity Volume (Å³)**	**Center Coordinates (x, y, z) (Å)**	**Box Dimensions (x, y, z) (Å)**
C1	-7.4	1981	(10, 1, -27)	(25, 25, 25)
C2	-6.0	583	(11, -3, -4)	(25, 25, 25)
C3	-6.3	317	(15, -10, -17)	(25, 25, 25)
C4	-6.3	242	(31, -2, -29)	(25, 25, 25)
C5	-5.9	95	(31, -4, -22)	(25, 25, 25)

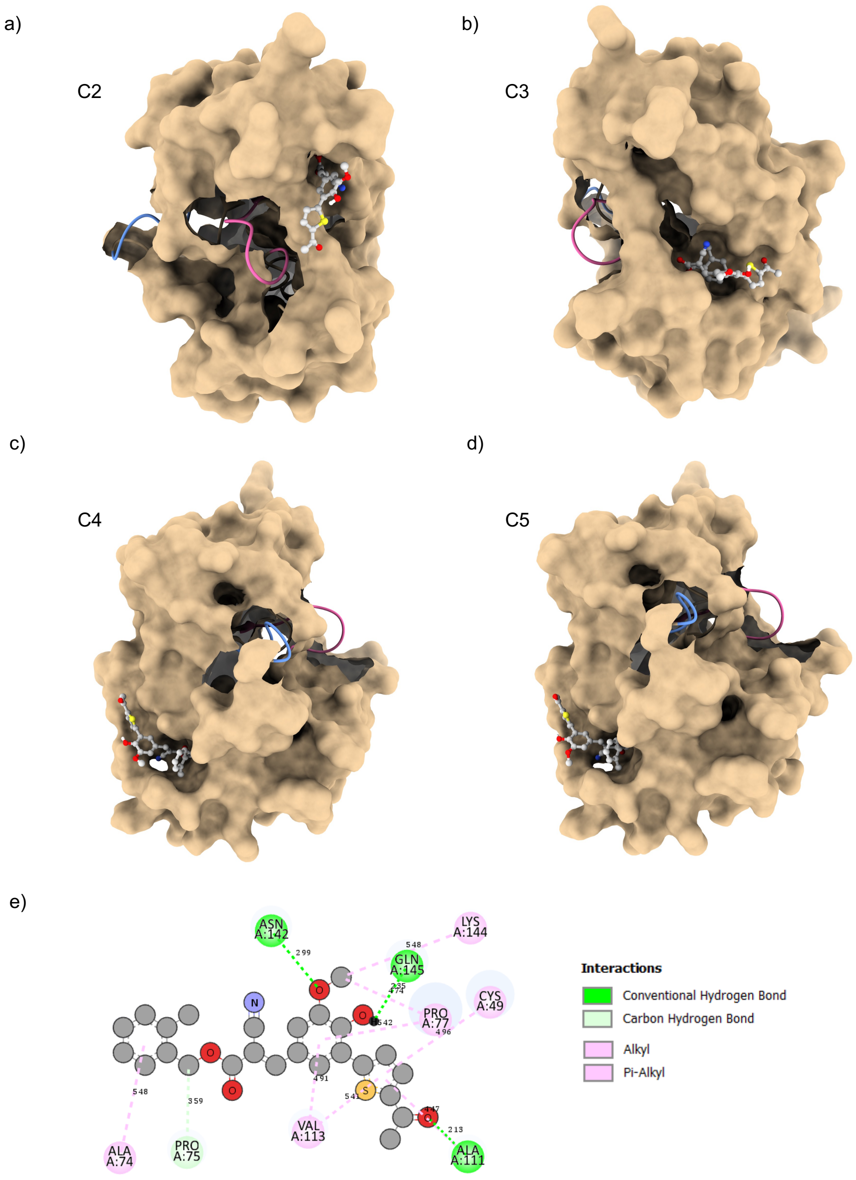

Supporting Information Figure S4. Computational docking of *PRLthiophenib* (7h) to the structure of PRL-3. (a-d) Docked Poses of *PRLthiophenib* in the alternative binding pockets C2-C5 of PRL-3 (PDB ID: 1V3A). The P-loop (pink, residues 103–110) and WPD-loop (blue, residues 68–72) provide structural context for the active-site environment. *PRLthiophenib* is displayed in ball-and-stick format, highlighting its spatial orientation within each cavity. Although the binding affinities in C2–C5 are weaker than in C1, these poses provide insight into potential alternative binding sites in PRL-3. (e) 2D Interaction Diagram of *PRLthiophenib* in the C1 Pocket of PRL-3 (PDB ID: 1V3A). This schematic, generated in Discovery Studio Visualizer, depicts the highest-scoring docked pose of *PRLthiophenib* (gray circles) within the C1 pocket of PRL-3. Conventional hydrogen bonds (green dashed lines) are observed with Asn142, Gln145, and Ala111, while additional carbon hydrogen bonds (light green dashed lines) and alkyl/π-alkyl interactions (pink dashed lines) involve Pro75, Ala74, Val113, Cys49, Pro77, and Lys144, respectively. The sulfur atom of *PRLthiophenib* (shown in yellow) lies in proximity to Cys104 (though not explicitly labeled in this 2D diagram), consistent with 3D analyses in ChimeraX indicating a potential interaction critical to PRL-3 function. Collectively, these hydrogen bonding and hydrophobic contacts help stabilize the ligand within the enzyme's active site. These results complement the 3D docking results and provide a clearer picture of how *PRLthiophenib* may modulate PRL-3 activity.

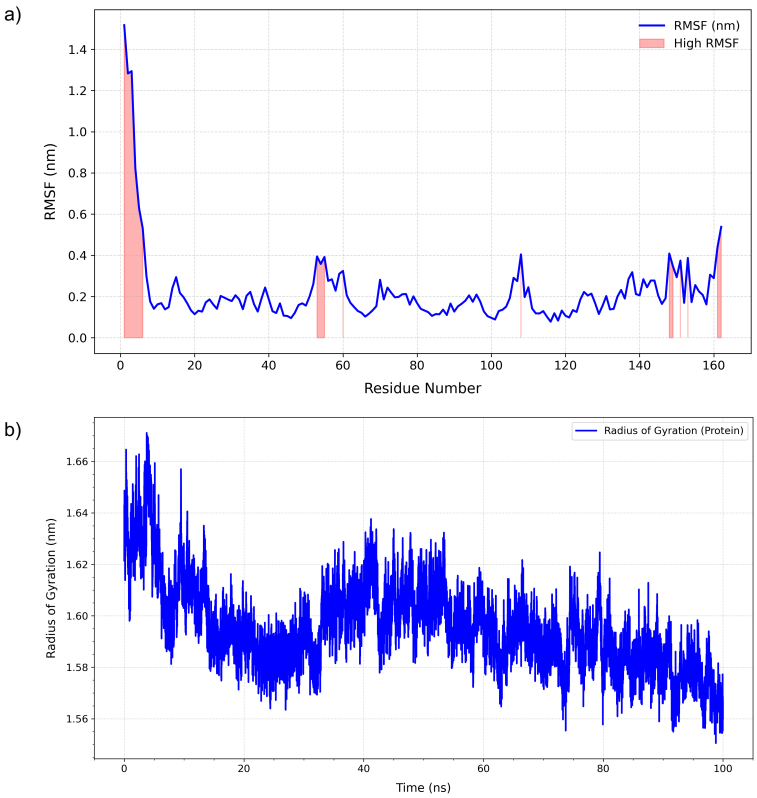

Supporting Figure S5. Molecular Dynamics Simulations of *PRLthiophenib* binding to PRL-3. (a) Root Mean Square Fluctuation (RMSF) of protein residues, depicting the RMSF (in nm) for each residue over the 100 ns molecular dynamics simulation, with residue number on the x-axis. The blue line represents the mean fluctuation magnitude per residue, whereas pink shading highlights areas of especially high RMSF. Elevated values at the *N*-terminus and near the *C*-terminus reflect typical terminal flexibility, while a smaller peak around residue 60 indicates a dynamic loop region. By contrast, most residues in the protein's core exhibit RMSF values below 0.4 nm, suggesting a stable secondary and tertiary structure. (b) Radius of gyration (Rg) of protein over time showing the Rg (in nm) for the protein throughout the 100 ns molecular dynamics simulation, with time (ns) on the x-axis. The Rg decreases from an initial value of roughly 1.66 nm to approximately 1.58 nm, suggesting that the protein adopts a more compact conformation as the simulation proceeds. Although minor oscillations persist (consistent with normal thermal motions), the overall Rg remains within a relatively narrow range, indicating stable protein folding.

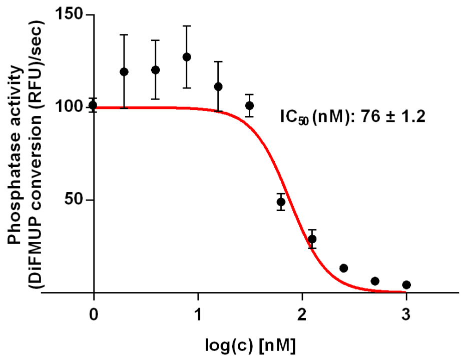

Supporting Information Figure S6. Sigmoidal curve for inhibition of JMS-053 against PRL-3.
Experiment was carried out in three independent experiments in triplicates. Results are depicted as mean ± SEM. IC50s were calculated using GraphPad Prism6 software, Nonlinear regression: log(inhibitor) vs. response - Normalized slope (four parameters).

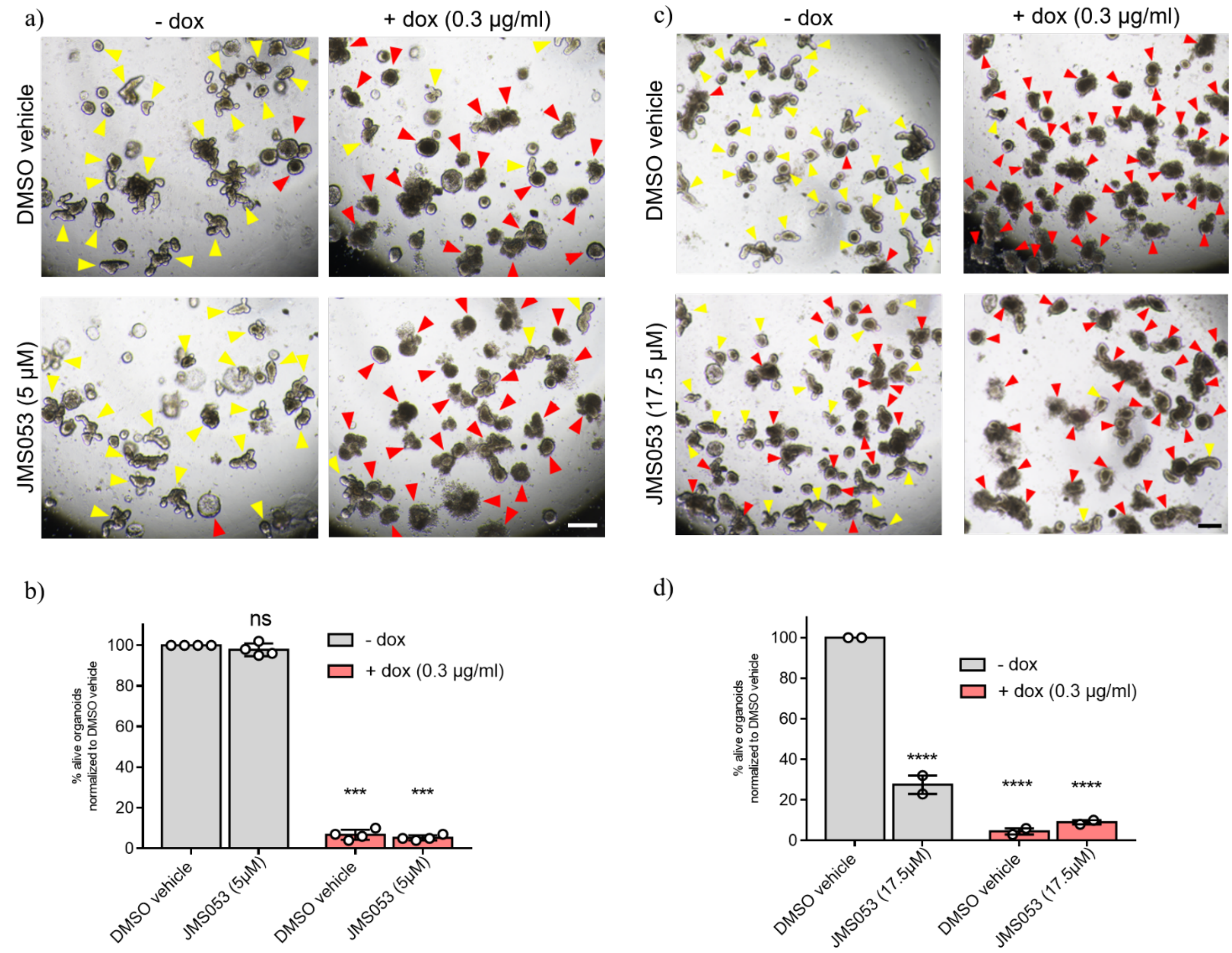

Supporting Figure S7. JMS-053 does not rescue the apoptotic effect induced by PRL-3 expression in SI organoids. Mouse derived small intestine (SI) organoids containing heterozygous HA-PRL-3 were grown for three days. PRL-3 expression was then induced by doxycycline (dox, 0.3 µg/ml). At the same time SI organoids were treated with either 5 µM or 17.5 µM JMS-053 and imaged after 24 hours. (a,c) Representative bright field images after 24 h of dox and compound or DMSO vehicle-treated SI organoids indicating dying (red arrows) and alive SI organoids (yellow arrows). Scale bar: 200 µm. (b) Quantification of counted alive SI organoids at 5 µM JMS-053. Results are depicted as mean ± SEM. Two-way ANOVA test was performed for $p < 0.05$. ns: not significant. Experiments were carried out in two independent experiments with SI organoids derived from two mice (round data points), all in technical triplicates. (d) Quantification of counted alive SI organoids. Results are depicted as mean ± SEM. Two-way ANOVA test was performed for $p < 0.05$. ns: not significant. Experiment was carried out in one independent experiment with SI organoids derived from two mice (round data points), all in technical triplicates.

Supporting Information Figure S8. ^{1}H-NMR and ^{13}C-NMR spectra of Analog 3 and hit compounds.

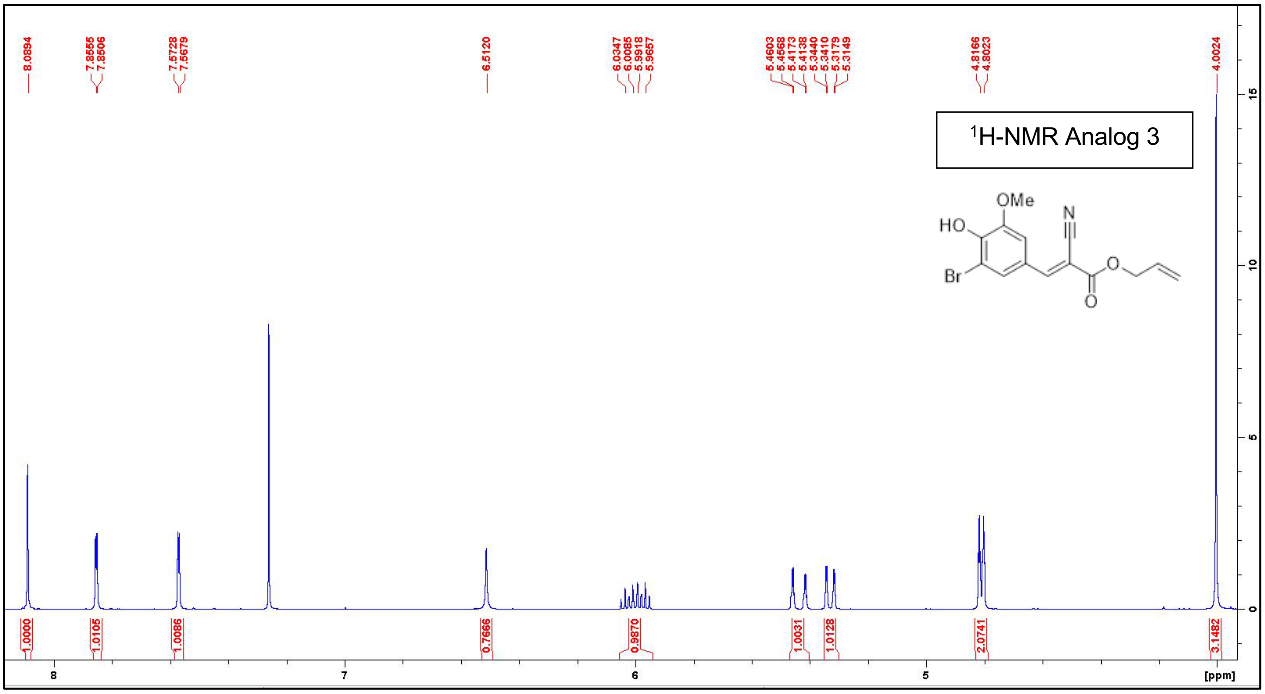

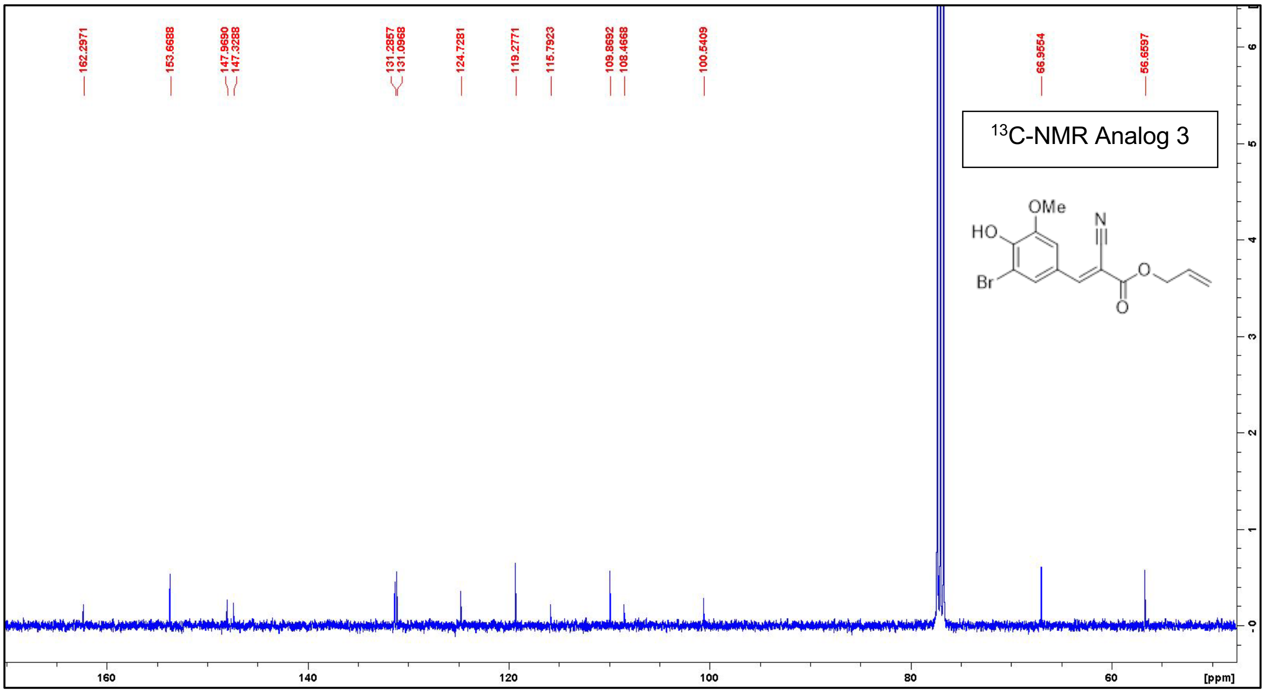

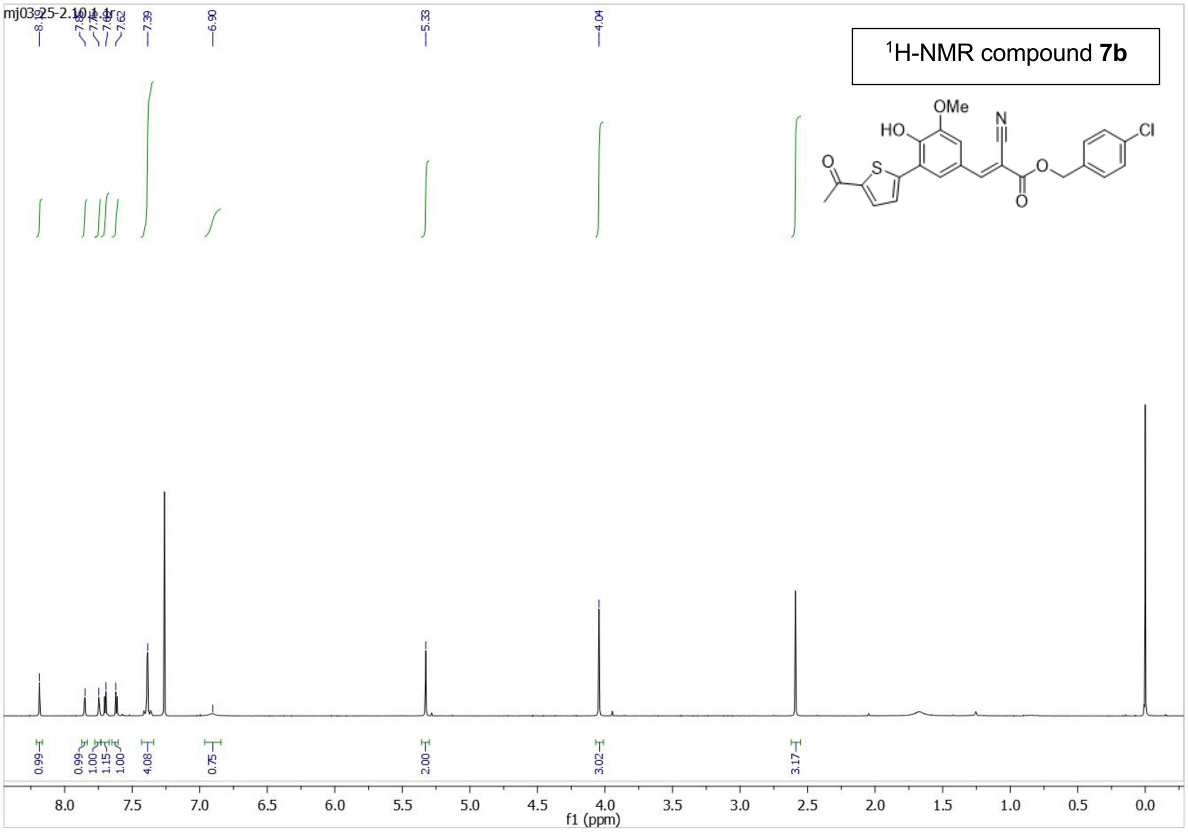

1H-NMR compound 7b
7.39
6.90
5.33
4.04
OMe
HO
N
O
S
O
O
Cl
0.99
0.99
1.00
1.15
1.00
4.08
0.75
2.00
3.02
3.17
8.0 7.5 7.0 6.5 6.0 5.5 5.0 4.5 4.0 3.5 3.0 2.5 2.0 1.5 1.0 0.5 0.0
f1 (ppm)

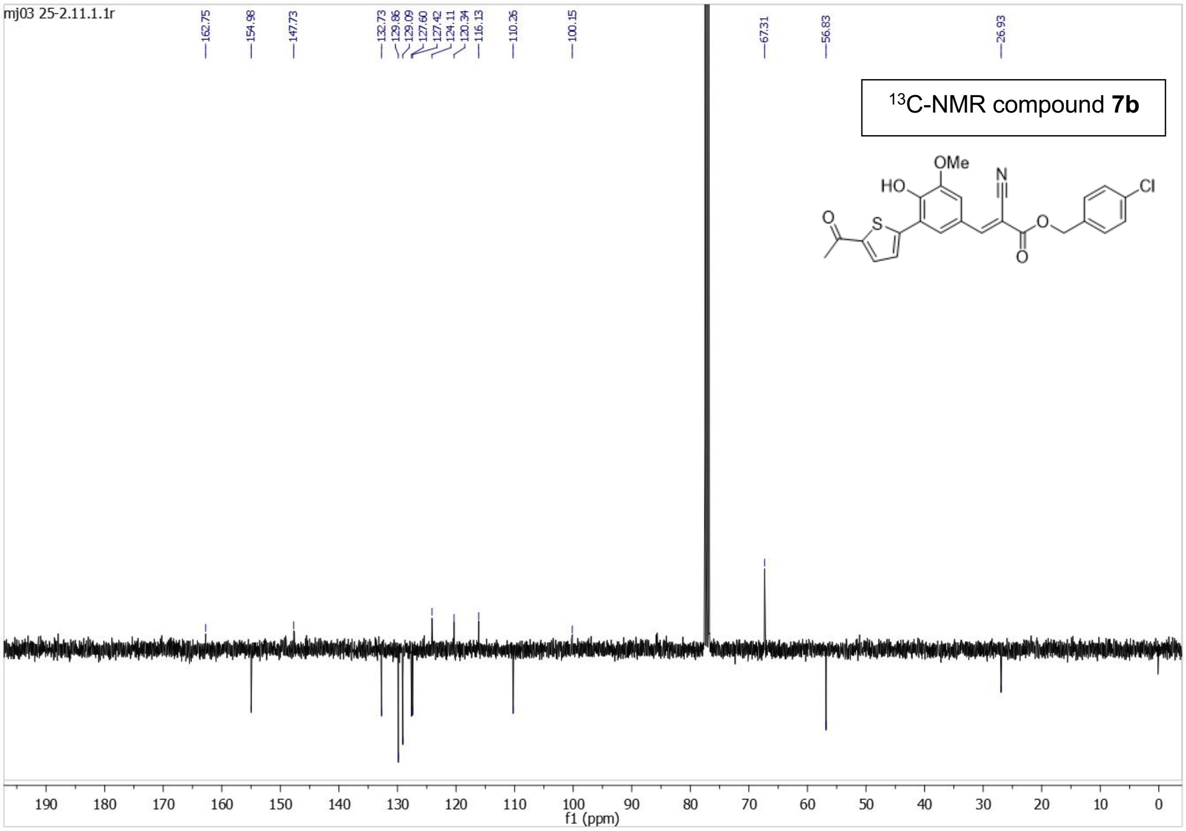

mj03 25-2.11.1.1r
162.75
154.98
147.73
132.73
129.86
129.09
127.60
127.42
124.11
120.34
116.13
110.26
100.15
67.31
56.83
26.93
13C-NMR compound 7b
OMe
HO
N
O
S
O
O
Cl
190 180 170 160 150 140 130 120 110 100 90 80 70 60 50 40 30 20 10 0
f1 (ppm)

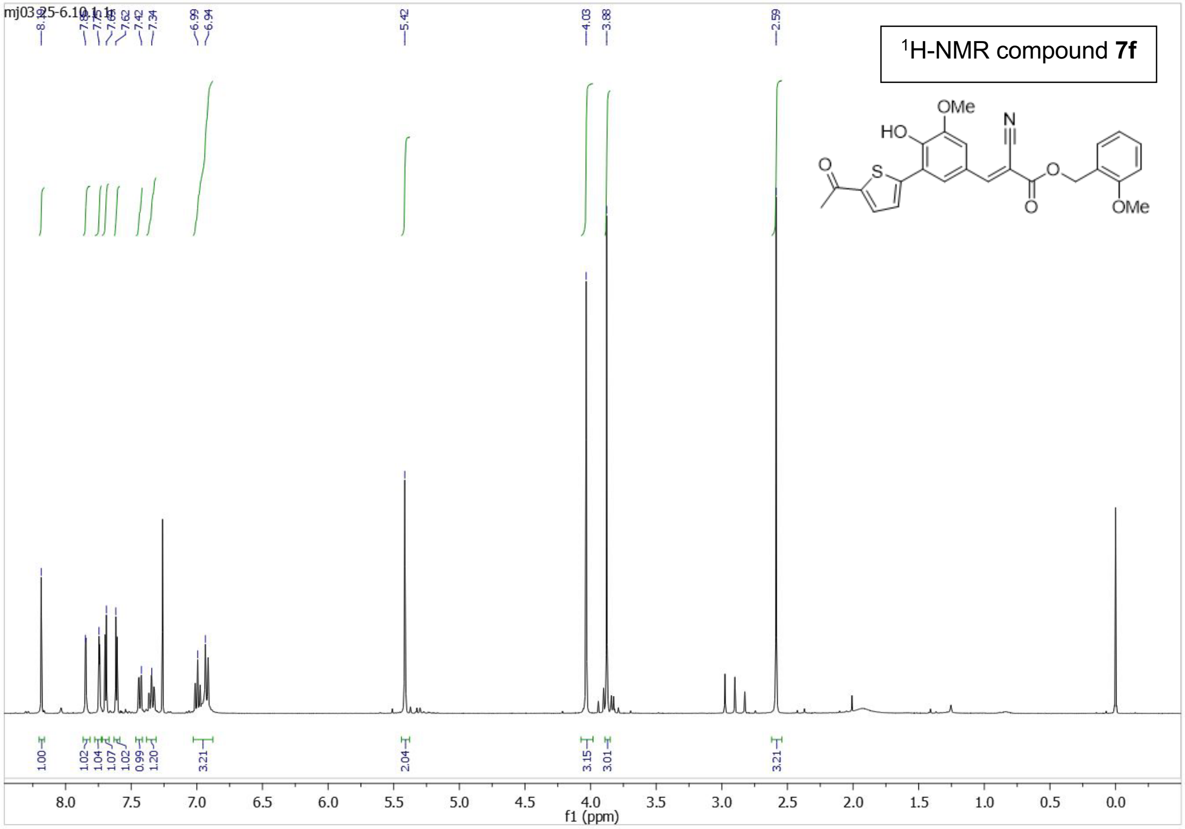

mj03 25-6.10.1.1r
1H-NMR compound 7f
OMe
HO
N
S
O
O
O
OMe
f1 (ppm)

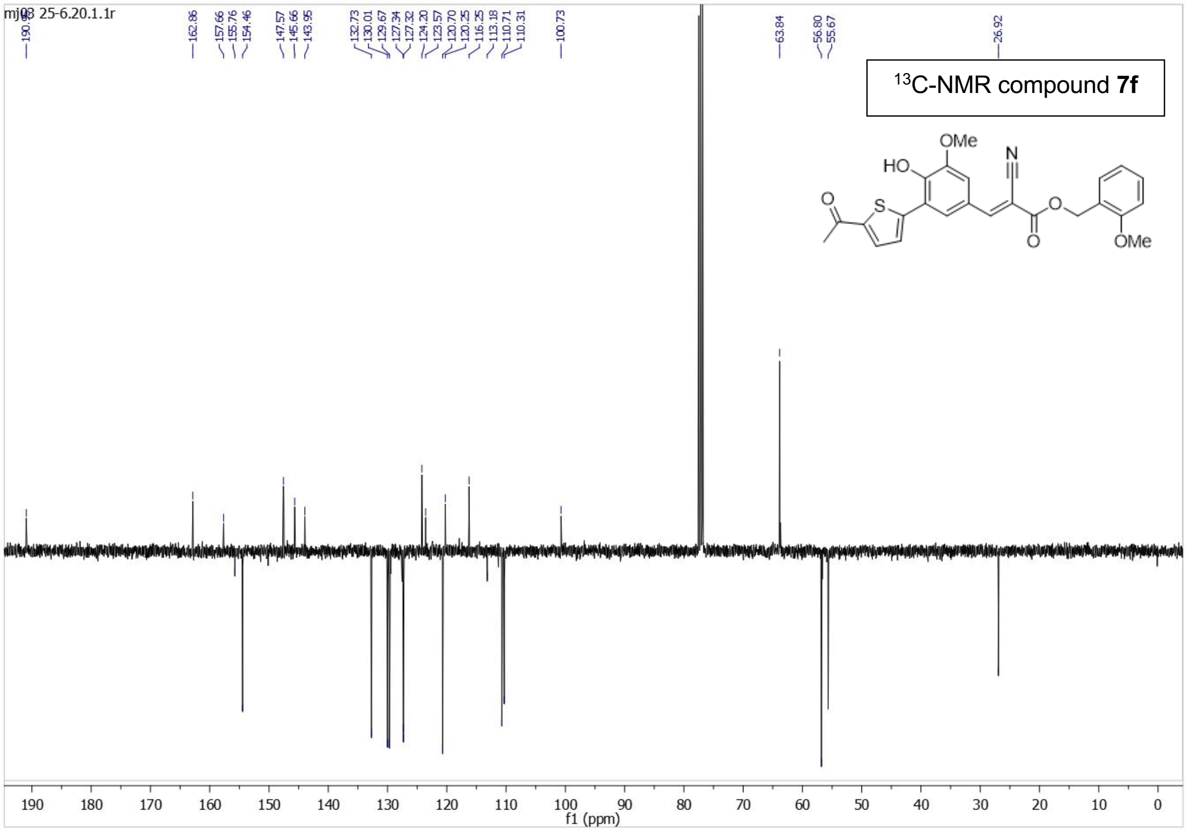

mj03 25-6.20.1.1r
13C-NMR compound 7f
OMe
HO
N
S
O
O
O
OMe
f1 (ppm)

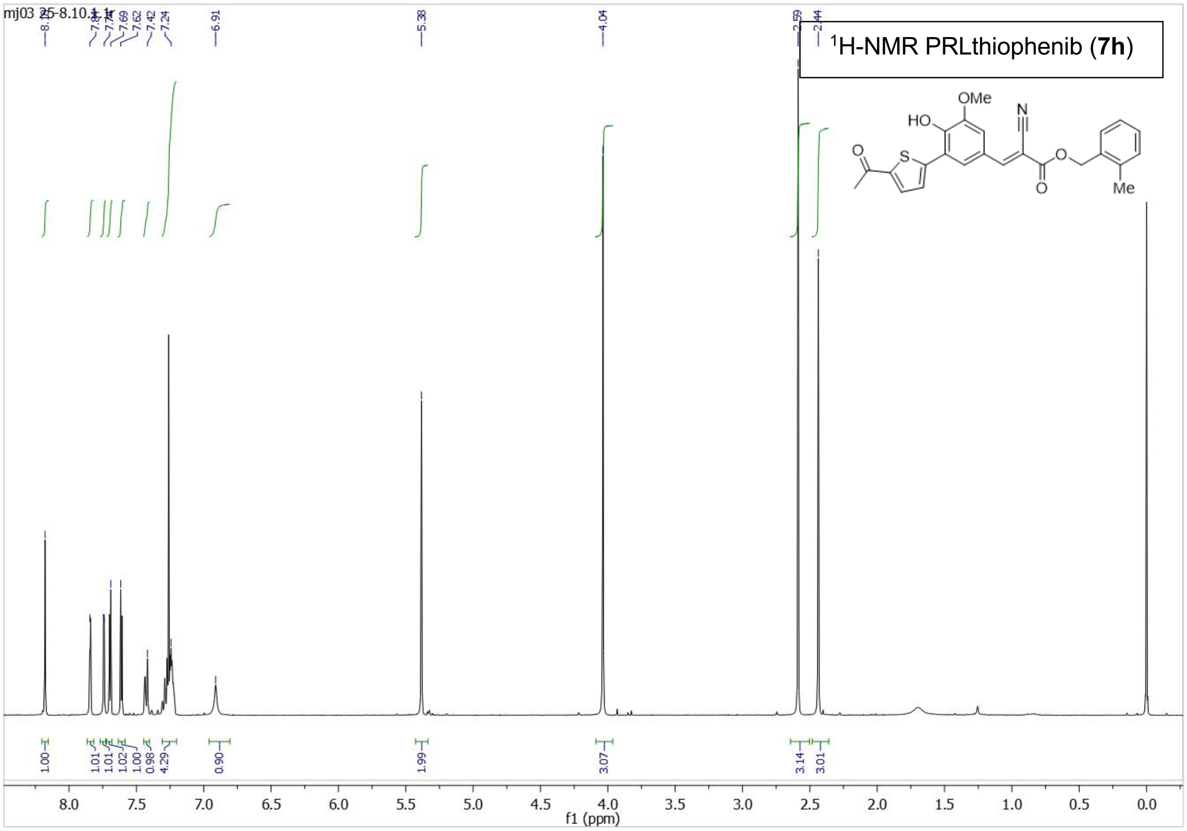

mj03 25-8.10.1.1r
1H-NMR PRLthiophenib (7h)
OMe
HO
N
S
O
O
O
Me
1.00
1.01
1.01
1.02
1.00
0.98
4.29
0.90
1.99
3.07
3.14
3.01
f1 (ppm)

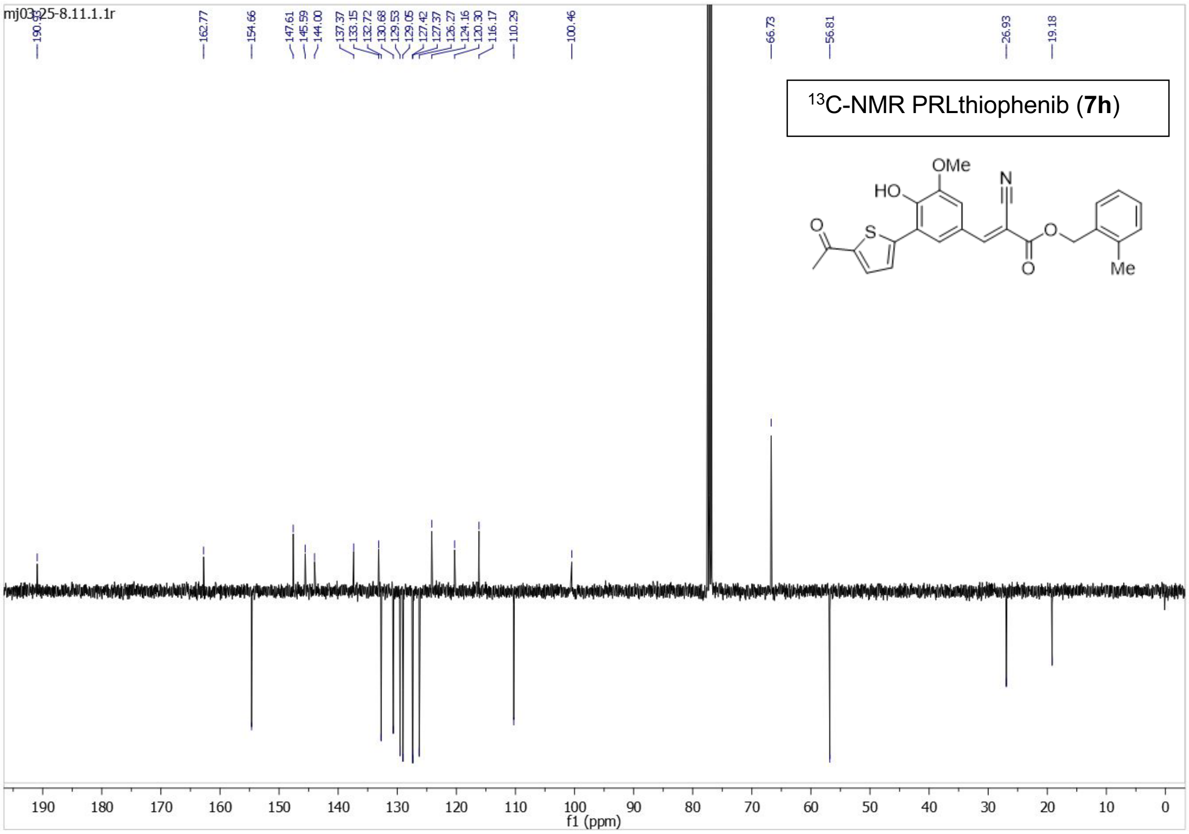

mj03 25-8.11.1.1r
162.77
154.66
147.61
145.59
144.00
137.37
133.15
132.72
130.68
129.53
129.05
127.42
127.37
126.27
124.16
120.30
116.17
110.29
100.46
66.73
56.81
26.93
19.18
13C-NMR PRLthiophenib (7h)
OMe
HO
N
S
O
O
O
Me
f1 (ppm)

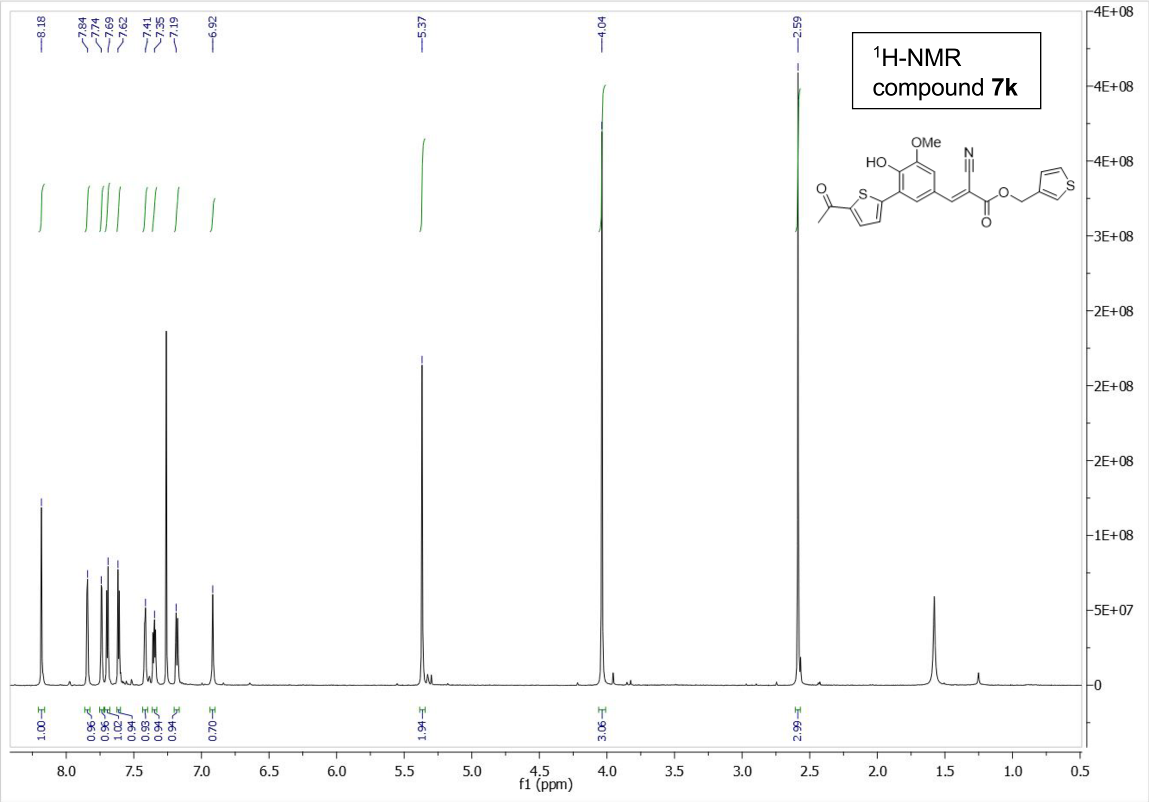

¹H-NMR compound 7k
8.18
7.84
7.74
7.69
7.62
7.41
7.35
7.19
6.92
5.37
4.04
2.59
OMe
HO
N
O
S
O
S
O
1.00
0.96
0.96
1.02
0.94
0.93
0.94
0.94
0.70
1.94
3.06
2.99
f1 (ppm)

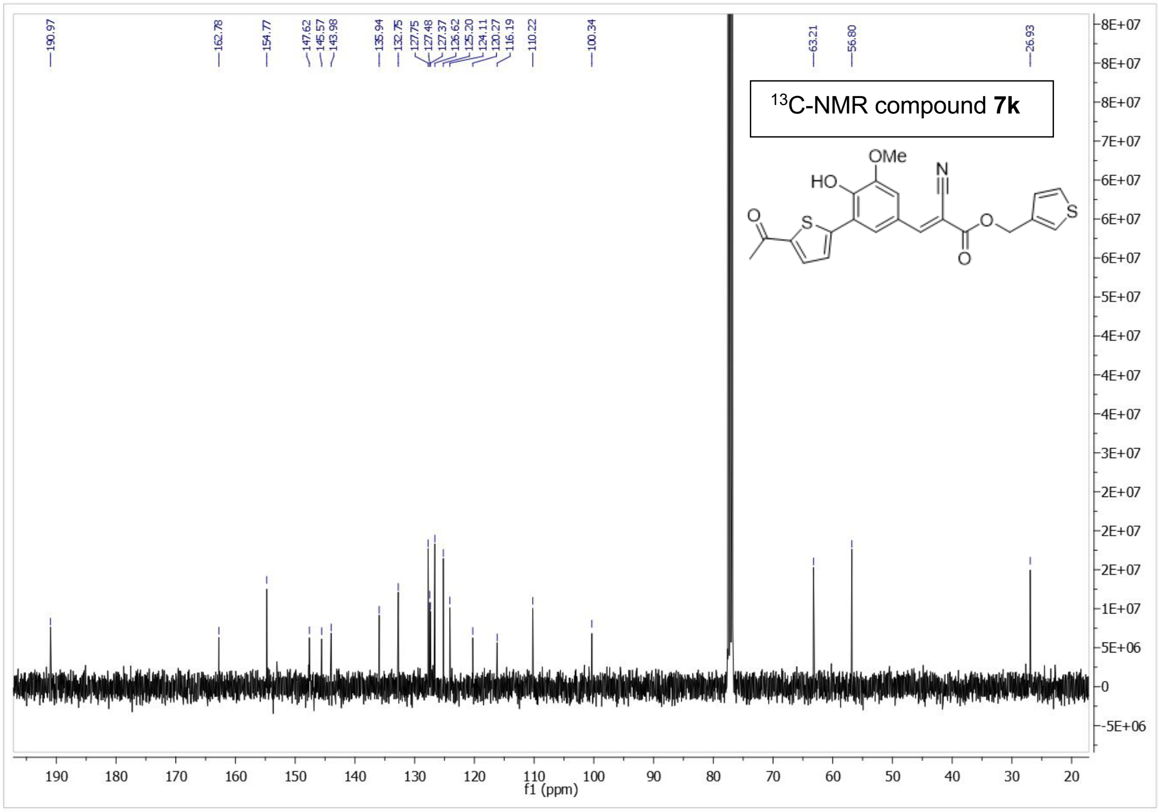

¹³C-NMR compound 7k
190.97
162.78
154.77
147.62
145.57
143.98
135.94
132.75
127.75
127.48
127.37
126.62
125.20
124.11
120.27
116.19
110.22
100.34
63.21
56.80
26.93
OMe
HO
N
O
S
O
S
O
f1 (ppm)

Supporting Information Figure S9. HPLC-MS spectra of Analog 3 and hit compounds.

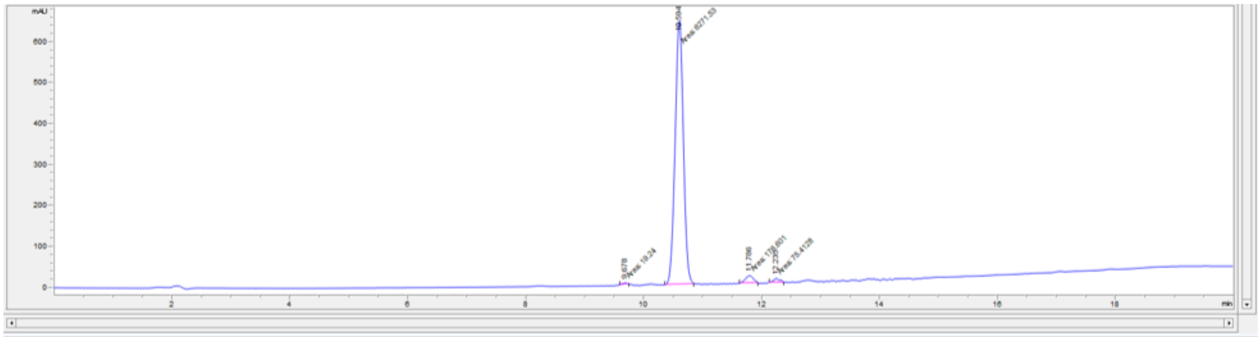

DAD1 A, at 230 nm for **Analog 3**
Purity: 95.84%

#	Time	Type	Area	Height	Width	Area%	Symmetry
1	9.678	MM	19.2	4.3	0.0738	0.294	0.999
2	10.594	MM	6271.5	644.2	0.1623	95.854	1.034
3	11.786	MM	176.6	18.8	0.1563	2.699	1.113
4	12.235	MM	75.4	10	0.1262	1.153	0.633

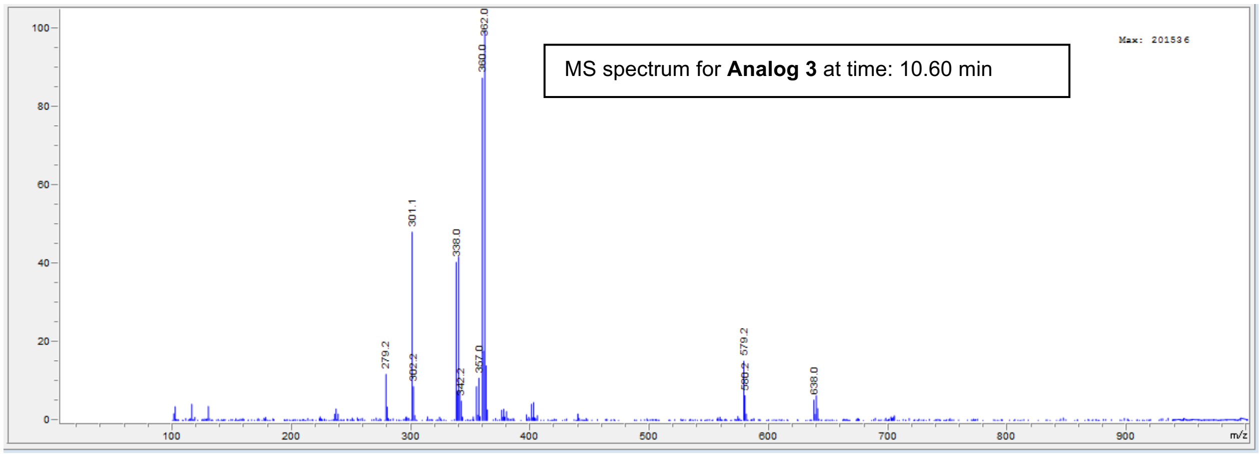

MS spectrum for **Analog 3** at time: 10.60 min

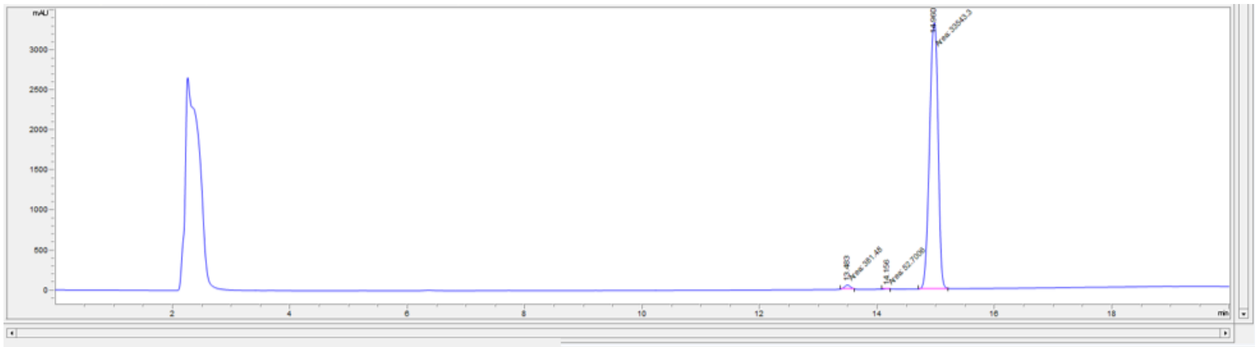

DAD1 A, at 230 nm for **compound 7b**
Purity: 98.72%

#	Time	Type	Area	Height	Width	Area%	Symmetry
1	13.483	MM	381.5	51.3	0.124	1.123	0.913
2	14.156	MM	52.7	7.8	0.1121	0.155	0.897
3	14.96	MM	33543.3	3334.6	0.1677	98.722	1.182

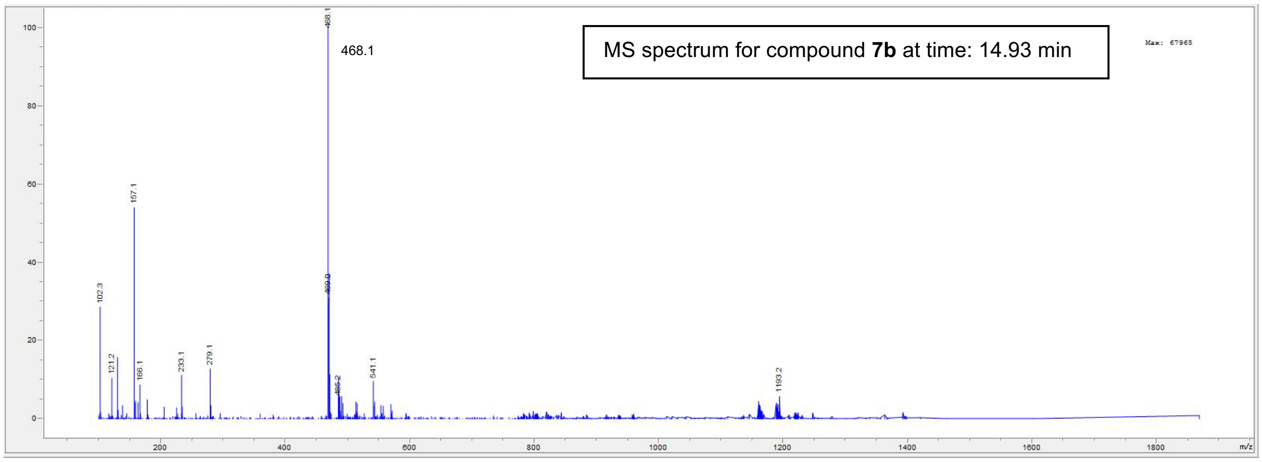

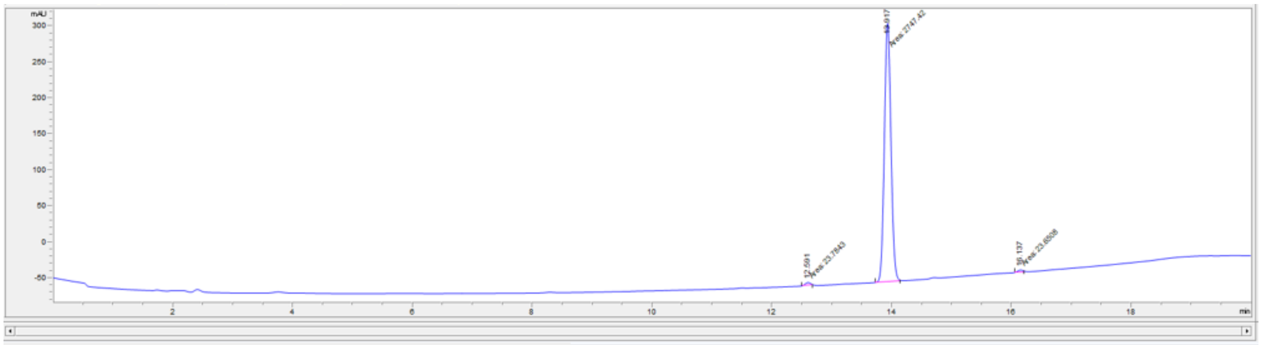

DAD1 A, at 230 nm for **compound 7f**
Purity: 98.30%

#	Time	Type	Area	Height	Width	Area%	Symmetry
1	12.591	MM	23.8	3.9	0.102	0.851	0.994
2	13.917	MM	2747.4	359.7	0.1273	98.303	0.921
3	16.137	MM	23.7	3.6	0.1107	0.846	0.99

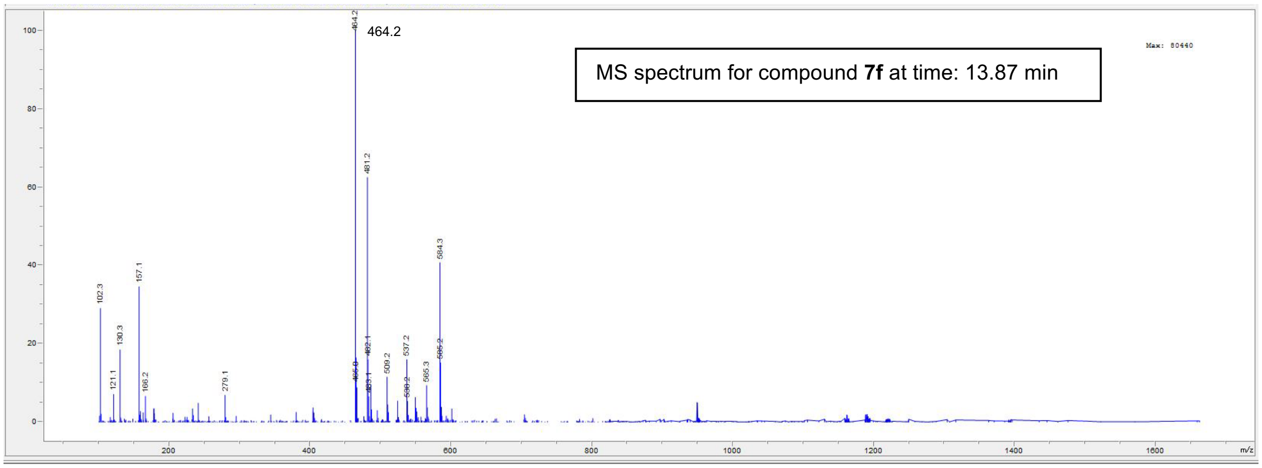

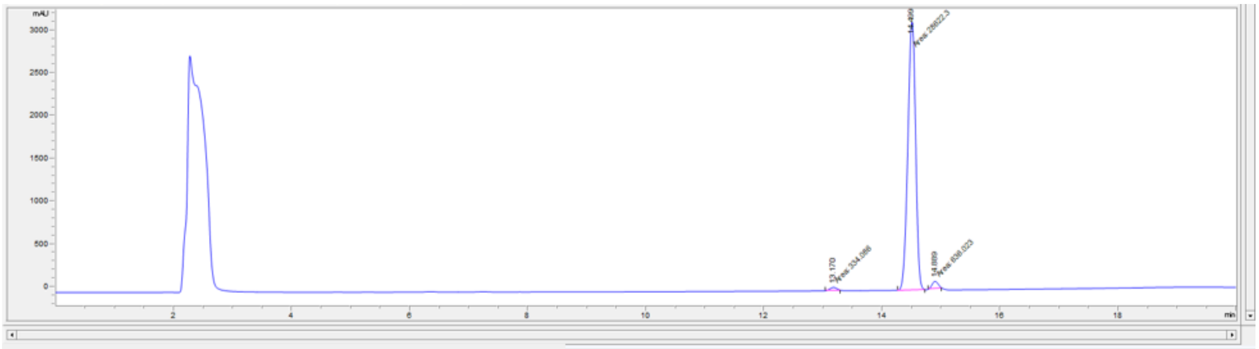

DAD1 A, at 230 nm for ***PRLthiophenib* (7h)**
Purity: 96.72%

#	Time	Type	Area	Height	Width	Area%	Symmetry
1	13.17	MM	334.1	40	0.1391	1.129	0.991
2	14.499	MM	28622.3	3130.2	0.1524	96.722	1.143
3	14.889	MM	636	87.1	0.1217	2.149	0.893

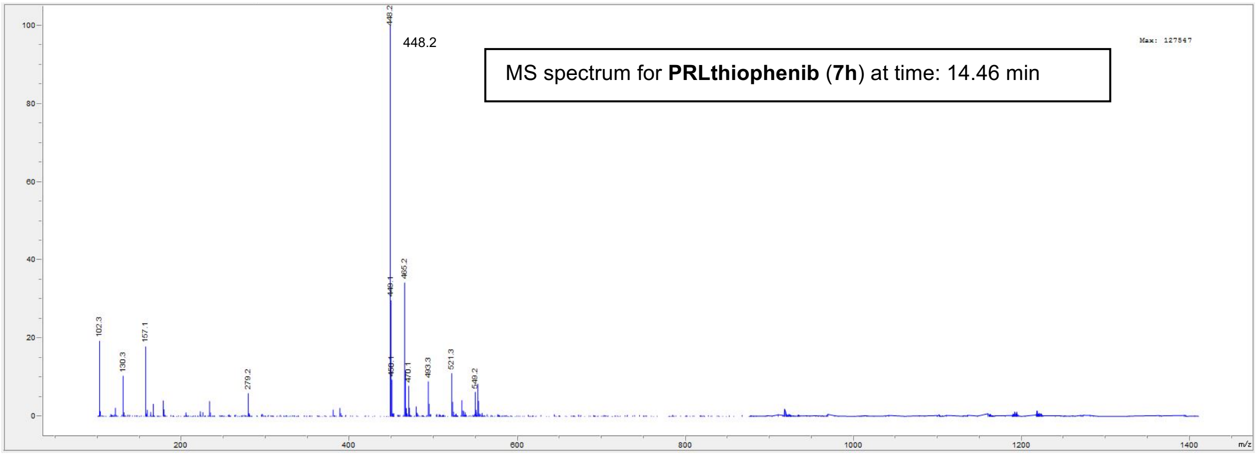

MS spectrum for **PRLthiophenib** (**7h**) at time: 14.46 min

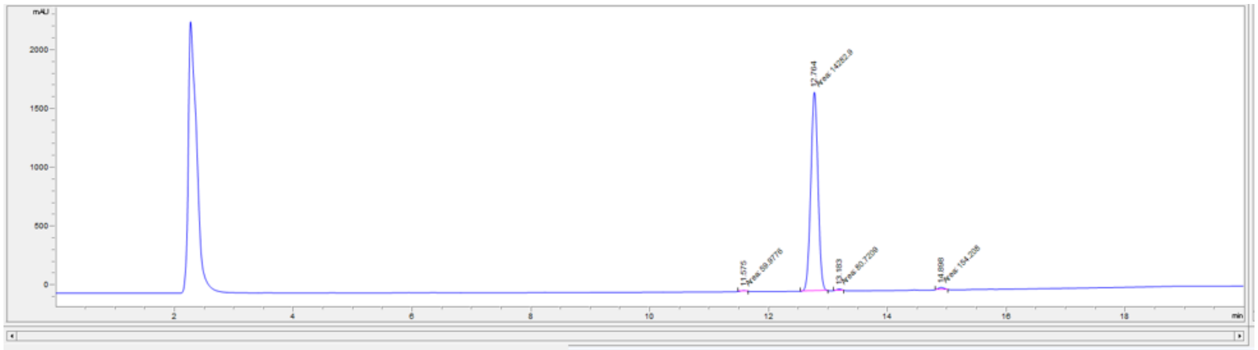

DAD1 A, at 230 nm for **compound 7k**
Purity: 97.98%

#	Time	Type	Area	Height	Width	Area%	Symmetry
1	11.575	MM	60	8.8	0.1136	0.411	1.037
2	12.764	MM	14282.9	1697	0.1403	97.977	1.001
3	13.183	MM	80.7	12.3	0.1096	0.554	0.946
4	14.898	MM	154.2	20.3	0.1264	1.058	0.807

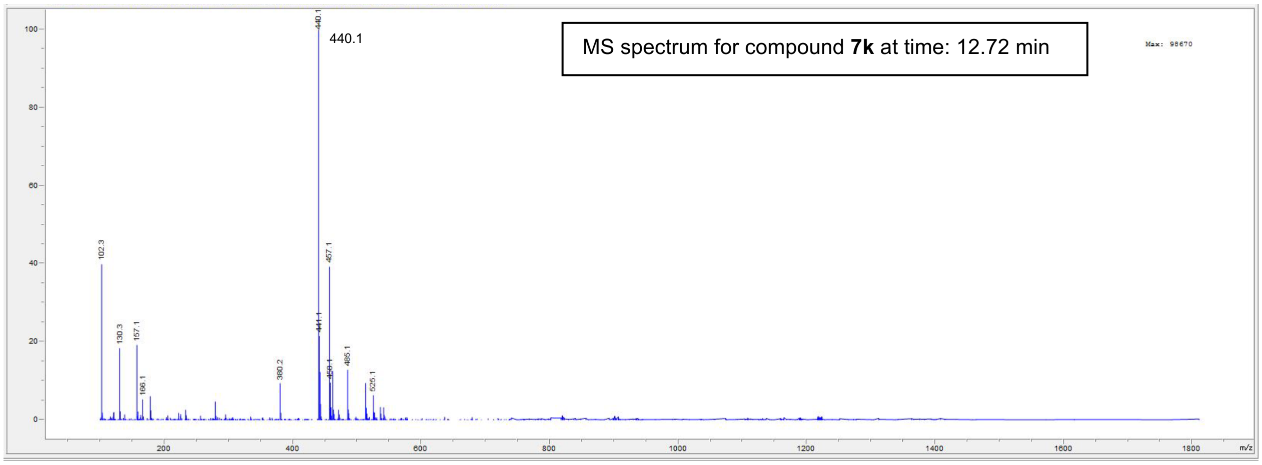

MS spectrum for compound **7k** at time: 12.72 min
